# Klotho in the kidney distal convolution regulates urinary Klotho excretion and kidney calcium reabsorption, but not phosphate homeostasis

**DOI:** 10.1016/j.kint.2026.01.030

**Published:** 2026-02-25

**Authors:** Laurent Bourqui, Adisa Trnjanin, Klaudia Kopper, Dominique Loffing-Cueni, Zsuzsa Radvanyi, Artyom Karpovich, Tara Rahimi, Rui Santos, Agnieszka Wengi, Johanne Pastor, Orson W. Moe, Johannes Loffing, Ganesh Pathare

**Affiliations:** 1 Institute of Anatomy, University of Zurich, Zurich, Switzerland; 2 Swiss National Centre of Competence in Research “Kidney Control of Homeostasis,” Zurich, Switzerland; 3 Charles and Jane Pak Center for Mineral Metabolism and Clinical Research, the University of Texas Southwestern Medical Center, Dallas, Texas, USA; 4 Departments of Internal Medicine and Physiology, the University of Texas Southwestern Medical Center, Dallas, Texas, USA; 5 Bone-Kidney Axis and Regeneration Laboratory, Department of Infectious Diseases and Public Health, Jockey Club College of Veterinary Medicine and Life Sciences, City University of Hong Kong, Hong Kong SAR

**Keywords:** distal convolution, FGF23, Klotho, TRPV5 and Ca^2+^ reabsorption

## Abstract

**Introduction:**

Klotho acts as a coreceptor for the phosphaturic hormone fibroblast growth factor-23 (FGF-23) and exists in both a membrane-bound and a soluble form (sKlotho) found in blood and urine. Klotho protein is moderately expressed in kidney proximal tubule and more abundant in the distal convolution (DC), which includes the distal convoluted tubule (DCT) and connecting tubule (CNT). However, the function of Klotho in the DC, particularly its role in sKlotho release and regulation of mineral metabolism, remains unclear.

**Methods:**

scRNA-seq was performed on isolated mouse DC cells. Four novel gene-modified mouse models were generated with Klotho deleted in the entire DC, the late DCT/CNT, the DCT only, and pan-tubular.

**Results:**

Using scRNA-seq on isolated mouse DC tubules, we showed that Klotho is more abundant in the late-DCT/CNT than in the early DCT. The composite data from three DC specific Klotho knockout mice support DC to be the primary source of urinary sKlotho, with 80% coming from the late-DCT/CNT and only 20% from the DCT. Notably, mice lacking Klotho in the entire DC (Kl-KO^DC^) maintained normal serum sKlotho, FGF-23, and phosphate homeostasis. Bulk RNA-seq of isolated fluorescent DC segments from Kl-KO^DC_Tomato^ mice revealed suppressed signaling by mitogen activated protein kinase and downregulation of several genes involved in kidney calcium ion handling (*Trpv5, Vdr, Pth1r, Klk1*). Consistently, the Kl-KO^DC^ mice exhibited profound hypercalciuria and reduced bone density. On the other hand, pan-tubular Klotho deficiency in mice led to severe phosphate imbalance and loss of both serum/urine sKlotho.

**Conclusions:**

DC-derived Klotho regulates urinary sKlotho levels and controls calcium ion reabsorption, while Klotho in proximal tubule maintains phosphate homeostasis and likely regulates circulating sKlotho levels.

α Klotho (Klotho) was discovered in mice as a gene that maintains healthspan. Mice with a hypomorphic Klotho gene show multiorgan degeneration including vascular calcification, osteopenia, sarcopenia, skin atrophy, pulmonary emphysema, and a shortened lifespan.^1^ In contrast, mice with transgenic Klotho overexpression have an extended lifespan and a resistance to aging-related conditions.^2^ Klotho has since been implicated in a wide range of functions, including regulation of mineral metabolism, ion transport, energy metabolism, as well as neuro-, cardio-, and nephroprotection.^3,4^ Moreover, Klotho deficiency has been associated with several disorders, including chronic kidney diseases, cardiovascular diseases, osteoporosis, and cognitive decline.^3,4^

Klotho is a transmembrane protein with a large extracellular region with 2 domains, KL1 and KL2, which share sequence homology with the β-glycosidase family.^3,5^ The extracellular domains can be cleaved by proteases and then shed from the plasma membrane to form soluble Klotho (sKlotho) that is found in blood, urine, and cerebrospinal fluid.^2^ Alternative splicing produces a nonsense mRNA transcript that is not translated into protein.^6^ Klotho is expressed in the kidneys, brain, and parathyroid glands with lower expression in a number of other organs.^1^ The kidneys are the main site of Klotho expression, as mice with a kidney-specific Klotho ablation have the same phenotype as global Klotho knockout mice.^7^ Within the kidney, Klotho expression is modest in the renal proximal tubule (PT)^8^ but high in the distal convolution (DC) that includes the distal convoluted tubule (DCT) and the connecting tubule (CNT).^1,8^

Membrane-bound Klotho and sKlotho (at supra-physiological concentrations in vitro) function as coreceptors for fibroblast growth factor 23 (FGF23), facilitating its binding to FGF receptors (FGFRs).^9^ Binding of FGF23 to the Klotho-FGFR complex activates the mitogen-activated protein kinase (MAPK) signaling pathway, which reduces PT phosphate (Pi) reabsorption by inhibiting the sodium-dependent Pi cotransporter (NaPi-IIa) in the PT, thereby promoting phosphaturia.^8,10-13^ The FGF23-FGFR-Klotho complex also suppresses the synthesis of 1,25-dihydroxyvitamin D (calcitriol) in the PT by decreasing the activity of CYP27b1 and increasing the activity of CYP24a1.^14,15^ Unlike membrane-bound Klotho, sKlotho is believed to have FGF23-independent endocrine or paracrine functions that help maintain mineral homeostasis by regulating NaPi-IIa in the PT, Ca^2+^ channel transient receptor potential vanilloid 5 (TRPV5), and K^+^ channel renal outer medullary potassium (ROMK) in the DC.^8,16-18^ Administration of recombinant sKlotho also has many beneficial effects on kidney and cardiovascular diseases.^19-22^

Studies using nephron segment–specific Klotho knockout mice in either the PT or DC suggested that the DC is the main source of sKlotho, and it was postulated that DC-derived sKlotho might contribute to the regulation of PT Pi transport in a paracrine manner.^23,24^ Olauson *et al.*^23^ used a Cdh16-Cre transgenic mouse line to delete Klotho in the DC, which caused hyperphosphatemia and resistance to FGF23; however, the mechanism was not studied. Notably, the original characterization of the *Cdh16*-Cre mice suggested that Cre is expressed not only in the DC but more broadly in nearly all renal epithelial cells, including the PT rendering the conclusion difficult.^25,26^ In line with these observations, others have used this cre-line for Klotho deletion in the entire kidney.^27^ In another study targeting Klotho expression in the PT, transgenic mice expressing Cre recombinase under the control of 3 different inducible promoters (PEPCK-Cre, Kap-Cre, and Slc34a1-Cre) were used to delete Klotho in the PT only.^24^ All 3 conditional Klotho knockout mouse models had an incomplete recombination with unchanged plasma sKlotho levels, which is difficult to interpret. Consistently, the mice had only mild or no hyperphosphatemia as long as they were not challenged with a high Pi diet. It was concluded that, when Klotho is deleted from the PT, sKlotho from the DC might contribute to regulating Pi reabsorption in the PT; however, this was not experimentally verified.^24^

In the present study, we used several genetically modified mouse lines harboring inducible and highly segment-specific deletion of Klotho in the DCT, the late-DCT/CNT, or the entire DC to show that the DC is the main source of urinary sKlotho but not serum sKlotho levels. In addition, we demonstrated that DC-derived Klotho plays an important role in Ca^2+^ homeostasis but not in Pi homeostasis.

## METHODS

### Animals

**Figure 1 ∣:**
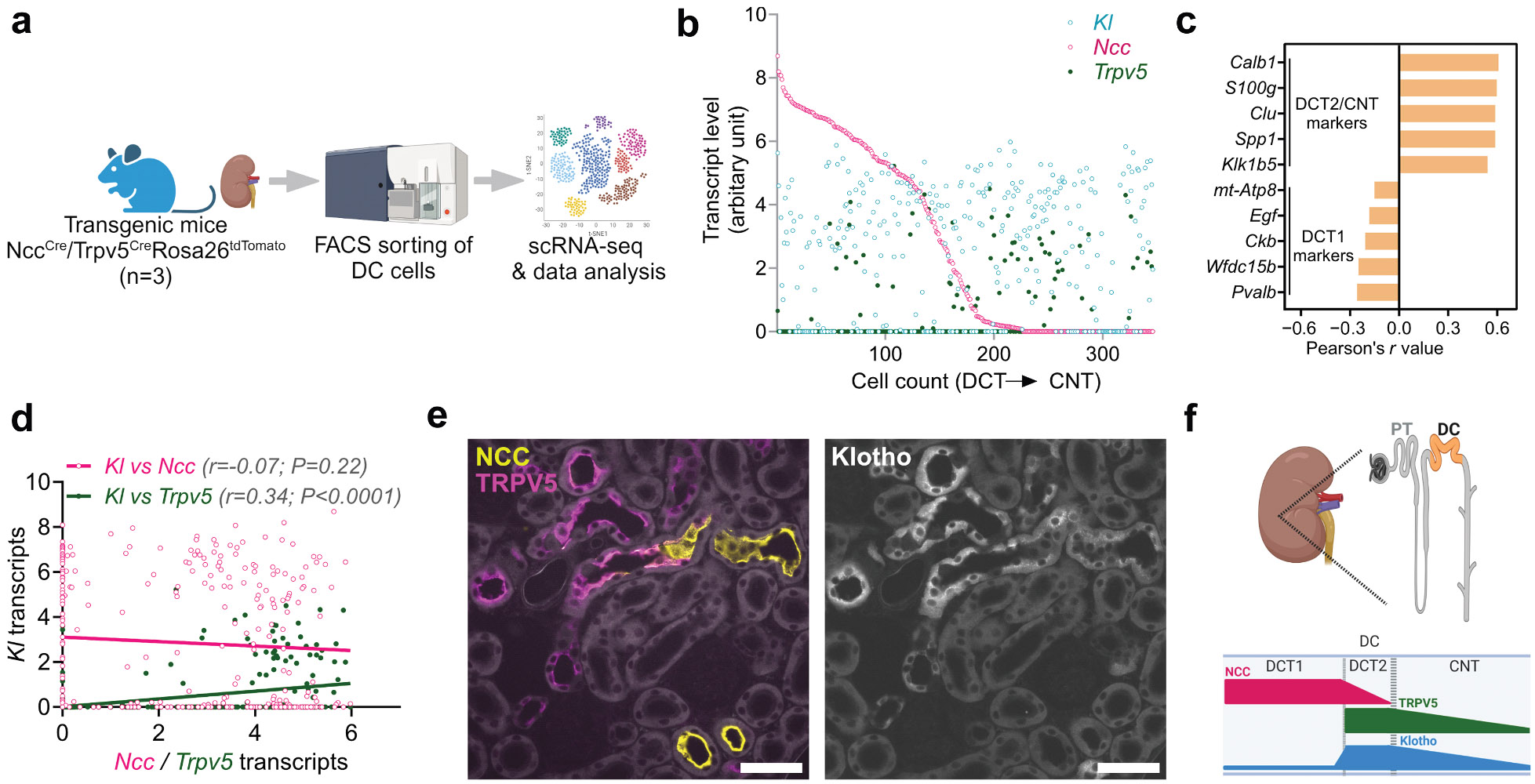
Klotho is enriched in the distal convoluted tubule (DCT)2/connecting tubule (CNT) with modest expression in DCT1. (**a**) Schematic diagram explaining the isolation of mouse DCT+CNT (distal convolution [DC]) cells and further single-cell RNA sequencing (scRNA-seq) analysis. (**b**) Transcript levels of *Kl*, *Slc12a3* (*Ncc*), and *Trpv5* across DC. Note the gradual decrease in *Ncc* marking the transition from DCT to CNT. (**c**) The top 5 positively and negatively correlated genes with *Kl* provided by bioinformatical correlation analysis (*P <* 0.0001). (**d**) Correlation plots depict no significant correlation between the DCT marker *Ncc* and *Kl*, while showing a statistically significant positive correlation between the DCT2/CNT marker *Trpv5* and *Kl*. (**e**) Immunofluorescence on consecutive kidney sections showing transient receptor potential cation channel subfamily V member 5 (TRPV5) in magenta and Na^+^ /Cl^−^ cotransporter (NCC) in yellow (left panel), whereas Klotho is shown in the right panel. Note the transition from DCT to CNT with changes in NCC and Klotho expression. (**f**) Based on these scRNA-seq and immunofluorescence data, within DC, Klotho is enriched in DCT2/CNT cells with modest expression in DCT1. FACS, fluorescence-activated cell sorting; PT, proximal tubule. To optimize viewing of this image, please see the online version of this article at www.kidney-international.org.

All mice were housed in the animal husbandry facility of the Laboratory Animal Service Center of the University of Zurich with free access to water and food *ad libitum*. All animal procedures were conducted in accordance with Swiss regulations and received approval from the veterinary administration of the Canton of Zurich (Kantonales Veterinäramt), Switzerland. The Trpv5-CreERT2, Ncc-CreERT2, and Klotho-flox mouse lines were custom-designed by Ozgene and maintained on a C57Bl/6 background. The Ncc-Cre mouse line has been characterized in a previous study.^28^ In the novel Trpv5^Cre^ line, the Cre-ERT2 sequence was introduced via homologous recombination into the 3′ -untranslated region of the *Trpv5* gene. In Klotho-flox strain, loxP sequences were inserted flanking exon 2. Cre-mediated deletion of the “floxed” exon introduced a translational frameshift, rendering downstream exons nonfunctional. The tdTomato mouse was obtained from the Jackson Laboratories Repository.^29^ For experimental breeding, homozygous cre mice (*Ncc^cre/cre^* and *Trpv5^cre/cre^*) were mated with heterozygous Klotho floxed (Klotho^fl/+^) and dtTomato (Tomato^tg/+^) as depicted in Figure 1. Doxycycline-inducible, renal-tubule specific Kl^fl/fl^/Pax8-rTA/LC1 or control (Kl^+/+^ /Pax8-rTA/LC1) mice were generated by breeding Kl^fl/fl^ and Pax8-rTA/LC1 mice.^30^ Offspring from these matings were then grouped based on age and sex for experimentation. Na^+^ /Cl^−^ cotransporter (NCC) knockout mice were described previously^31^ and were maintained on a standard chow diet. The mouse models used in this study are summarized in Supplementary Table S1. Biopsies were obtained from the toes and subjected to digestion in 25 mM NaOH heated to 95 °C for 1 hour. The reaction was stopped by the addition of 1 M Tris-HCl pH8. Genomic DNA extracts were then amplified by polymerase chain reaction (PCR) using the corresponding primers listed in Supplementary Table S2.

#### Characterization of Trpv5^Cre^ mice.

Adult male wild-type, Trpv5^Cre/+^ and Trpv5^Cre/Cre^ mice, aged 8–10 weeks, were treated with tamoxifen (2 mg of tamoxifen/mouse/d) for 5 days. Two weeks after the last tamoxifen administration, the mice were placed under deep anesthesia (isoflurane 2%–4%; 0.5–0.6 l/min; buprenorphine 0.1–0.2 mg/kg, s.c.), and blood was withdrawn from the vena cava before perfusion with phosphate-buffered saline (PBS) and removal of the kidneys. Whole blood parameters were determined immediately (ABL-FLEX80; Radiometer). Kidneys were snap-frozen and further processed for protein analysis and quantitative reverse transcription PCR (qRT-PCR), respectively. In a different set of mice, kidneys were fixed with 3% para-formaldehyde/0.1M via retrograde perfusion through the aorta. The kidneys were then removed and frozen in liquid propane for further use. For histochemical analysis, 5 μm cryosections were incubated with primary antibodies (Supplementary Table S3) at 4 °C overnight. The binding sites of the primary antibodies were detected using fluorescently labeled secondary antibodies (Supplementary Table S3). Light microscopy of the sections was performed using a DM6000 (Leica).

#### Induction in mice.

Mice aged 6–8 weeks were administered 2 mg of tamoxifen per day via gastric gavage for 5 consecutive days. The tamoxifen was diluted in a solution consisting of 10% ethanol and 90% sunflower oil. After the tamoxifen induction, the mice underwent a resting period of 3 weeks before being included in the following experiment. On the other hand, doxycycline (2 g/l) and sucrose (20 g/l) were administered in tap water to both Kl-KO^Kidney^ and control mice, with the solution changed every second day.

#### Metabolic cage experiment.

The metabolic cages (Tecni-plast S.p.A.) were equipped with a cooling device that allows urine sampling at 4 °C. Between the ages of 8 and 15 weeks, the mice were housed individually in metabolic cages with *ad libitum* access to tap water and wet food. The standard laboratory chow (Ssniff; Spezialdiäten GmbH) was mixed with water at a 1:1 ratio. After a 2-day acclimatization period, 24-hour urine samples were collected. Moreover, other measurements such as body weight, food, and water consumption were also recorded.

### Blood and urine analysis

The mice were placed under deep anesthesia (isoflurane 2%–4%; 0.5–0.6 l/min; buprenorphine 0.1–0.2 mg/kg, s.c.), and blood was withdrawn from the vena cava. Immediately after collection, the samples were processed with the ABL825Flex Blood Gas Analyzer (Radiometer) to quantify the blood gas and ion concentrations. Subsequently, the serum was separated, transferred into Eppendorf tubes, and stored at −80 °C until further analysis. Urinary sKlotho levels were measured using a commercial enzyme-linked immunosorbent assay (ELISA) kit (IBL; Cat. #27601).^32^ Urine samples were diluted 50× to measure sKlotho by ELISA. In a separate experiment, urine was mixed with 2× Laemmli buffer, heated at 95 °C for 5 minutes, and loaded into gel electrophoresis normalized to amount of creatinine to perform immunoblotting for Klotho. Serum sKlotho levels were determined using the immunoprecipitation-immunoblotting approach,^33^ as previously described. Serum calcitriol levels (IDS; Cat. #AC-62F1), parathyroid hormone, intact-FGF23, and c-terminal FGF23 (Quidel; Cat. #60-2305, #60-6800, and #60-6300, respectively) were measured by ELISAs according to the manufacturer’s instructions. Urine and plasma Pi levels were determined at Zürich Integrative Rodent Physiology using SYNCHRON LX systems (Beckman Coulter) and UniCel DxC 600/800 systems (Beckman Coulter). Flame photometry with EFOX 5053 (Eppendorf) was used to measure levels of urinary Na^+^, K^+^, and Ca^2+^. Urine creatinine was assessed using the Jaffe method, and urine parameters were normalized to urinary creatinine.

### Micro–computed tomography of bone

The micro–computed tomography analysis was performed at Zürich Integrative Rodent Physiology, University of Zürich as described earlier.^34^ Briefly, dissected femurs were examined using a micro–computed tomography scanner (Quantum Fx; Perkin Elmer) equipped with a 5-mm focal size, a tube voltage of 90 kV, a tube current of 100 μA, and a voxel size of 10 μm. A calcium hydroxyapatite phantom (QRM GmbH) was also imaged with the same settings to calibrate the bone data to bone mineral density (BMD) values. All images were reconstructed and analyzed using Analyze 12.0 software (AnalyzeDirect, Inc). Analysis was conducted on a segment of 100 slices near the distal femoral growth plate, beginning where the epiphyseal cap structure ended. For the BMD calculations, average gray level intensities were recorded for the various inserts of the phantom, and a linear correlation was established between the gray level intensity and BMD values.

### Tissue homogenization

The snap-frozen organs were transferred into ice-cold tubes containing ceramic beads (32 Magna Lyser Green Beads; Roche) and lysis buffer. For protein extraction, we used a detergent-free lysis buffer consisting of 200 mM mannitol, 80 mM *N*-2-hydroxyethylpiperazine-*N*′ -2-ethanesulfonic acid, and 40 mM KOH, supplemented with protease inhibitor (Complete Ultra; Roche) and phosphatase inhibitor (PhosSTOP; Roche). As for RNA extraction, we used the lysis buffer provided by the extraction kit. Samples were homogenized using a Bead Mill Homogenizer (OMNI International).

### RNA extraction and quantitative PCR

For total RNA isolation from homogenized organs, the NucleoSpin RNA isolation (Macherry Nagel; Cat. #740955) was used according to the manufacturer’s protocol and as previously described.^35^ Equal concentrations of isolated RNA (500 ng) were reverse transcribed into cDNA using the high-capacity cDNA reverse transcription kit (Thermo Fisher; Cat. #4374966). The resulting cDNA was then subjected to qPCR with SybrGreen Master Mix (Roche; Cat. #4707516001) using primers, as listed in Supplementary Table S4. The relative quantification of gene expression through double-delta Ct (threshold cycle) was performed after normalization to *Tbp* expression.

### Immunoblotting

Immunoblot was performed as previously described.^35,36^ In short, protein concentrations were determined using the Bradford protein assay (Uptima; Cat. #UPF86421). Denaturation of proteins (30 μg) was performed in Laemmli buffer (10% sodium dodecylsulfate, 0.5 M Tris·HCl [pH 6.8], 0.5% bromophenol blue, and β-mercaptoethanol). Electrophoresis was performed on 8%–12% acrylamide gel to separate the proteins, which were then transferred to the nitrocellulose membrane (Bio-Rad). After protein transfer, membranes were blocked for 20–45 minutes with Odyssey Blocking solution (PBS diluted [1:1] at room temperature) and incubated with the primary antibody and β-actin diluted in Odyssey Blocking solution (LI-COR; Cat. # 927-70001) (PBS [1:1] supplemented with Tween 20 [1:1000] at 4 °C) overnight. The following day, after repeated washes with PBS, secondary antibodies (Supplementary Table S3) were applied to the membrane in Casein Blocking solution (Sigma-Aldrich), diluted in deionized water (1:10). After repeated washes in PBS, immunoreactive bands were visualized using the Odyssey IR imaging system (LI-COR Biosciences). Signal densities were quantified with ImageJ S5 and normalized to β-actin.

### Immunofluorescence

Perfusion-fixed kidneys underwent slicing into 0.5-mm-thick sections, which were mounted on cork plates and rapidly frozen at −80 °C in liquid propane before storage at the same temperature. From the specimen, 4-μm-thick sections were cut using a cryostat (Microm HM 550; Thermo Fisher) and placed onto Superfrost slides (Epredia). After blocking with normal goat serum (diluted 1:10 in 2% bovine serum albumin in PBS) for 30 minutes, sections were incubated overnight at 4 °C with primary antibodies (refer to Supplementary Table S3) diluted in 1% bovine serum albumin in PBS within a humid chamber. After rinsing in PBS, sections were exposed to fluorescent dye-conjugated secondary antibody diluted in 1% bovine serum albumin in PBS supplemented with 4′,6-diamidino-2-phenylindole (1:1000) for 2–3 hours at room temperature in a humid chamber in the dark. After the final washing in PBS, coverslips were mounted using DAKO Glycergel Mounting Medium (Agilent). Kidney sections were analyzed using a Leica DM6000 B fluorescence microscope, and image capture was performed using a Leica DFC350 FX fluorescence monochrome digital camera (Leica Microsystems). Image processing was conducted using ImageJ S5.

### RNAscope experiments

For the detection of *Kl* mRNA in the kidney, frozen 4-μm-thick sections were cut using a cryostat, fixed in 4% formaldehyde/PBS, and processed according to the manufacturer’s instructions (ACDBio). The commercially available Klotho RNAScope probe (RNAscope Probe-Mm-Klb-O1, Cat. No. 481211) was incubated with the samples for 2 hours as per the manufacturer’s recommendations. After the assay, samples were fixed once more in 4% formaldehyde/PBS, and an immunofluorescence assay for NCC was performed as described above.

### Single-cell sorting and SMARTseq2 sequencing

#### Tissue preparation and single-cell sorting.

Male Ncc-Cre/TdTomato and Trpv5-Cre/TdTomato mice were induced by tamoxifen at the age of 6 weeks, and the kidneys were retrieved at the age of 8 weeks. In total, we used 6 male mice (3 male Ncc-creERT2-TdTomato and 3 male Trpv5-creERT2-TdTomato) to isolate single cells from DCT1, DCT2, and CNT. Briefly, mice were perfused with cold PBS containing hyaluronidase, DNase, and collagenase, minced and incubated at 37 °C for 45 minutes. The cell suspension was filtered through 40 μm pored nylon filters and counter-stained with 4′,6-diamidino-2-phenylindole for live/dead discrimination. Cells were sorted with FACS Aria III 5L with an 85 μm nozzle. Dead cells were excluded based on 4′,6-diamidino-2-phenylindole staining. Doublets were excluded from the sorting procedure based on pulse width. Single kidney cells were sorted directly into a 384-well plate containing 10 μl of lysis solution and barcoded poly(T) reverse-transcription primers. To ensure cell viability, we prepared and sorted 2 mice per day and isolated 192 single cells.

#### Smart-Seq2 library preparation and sequencing.

The libraries were prepared using a miniaturized version of the Smart-Seq2 protocol with a TTP Labtech Mosquito HV pipetting robot. Briefly, single cells were directly sorted into 384-well plates containing 0.8 μl of lysis buffer (0.1% vol/vol Triton X-100, 2.5 mM deoxynucleotide triphosphates, 2.5 μM oligo-deoxythymidine, 1 U/μl Promega RNasin Plus RNase inhibitor). Reverse transcription was performed in a final volume of 2 μl, followed by cDNA amplification in a final volume of 5 μl. The quality of the cDNAs was evaluated using an Agilent 2100 Bioanalyzer. cDNA (0.1 ng) from each cell on the plate was individually prepared in the Illumina Nextera XT kit in a final volume of 2 μl, followed by barcoding and library amplification in a final volume of 5 μl. The resulting 384 libraries were pooled, double-sided size selected (0.5× followed by 0.8× ratio using Beckman Ampure XP beads), and quantified using an Agilent 4200 TapeStation System. The pool of libraries was sequenced in an Illumina HiSeq2500 with a depth of around 500,000 reads per cell (around 200 million reads per plate). The standard analysis procedure and quality control were performed with the Seurat package.^37,38^

#### Single-cell transcriptome data analysis.

The single-cell RNA sequencing (scRNA-seq) reads were mapped to Ensembl Mus musculus reference (release 91) with STAR (v2.6.1c) aligner^39^ and counted with featureCounts in Rsubread (v1.32.4).^40^ Then the R package Seurat (v2.3.4)^37^ was used for downstream data analysis. To ensure that low-quality cells did not distort the analysis results, we filtered the cells that expressed fewer than 500 genes and used a library size smaller than 50,000. We also discarded genes that were detected in fewer than 5 cells, resulting in 14,457 genes. Then “LogNormalize” function from Seurat was used to normalize the gene expression into log-scale with a scale factor of 100,000. A total of 1914 highly variable genes were selected by “FindVariableGenes” with parameters “x.low.cutoff = 0.1, x.high.cutoff = 8, and y.cutoff = 1.” After removing unwanted cell-specific bias of library size and percentage of mitochondrial reads, we performed principal component analysis to reduce the dimensionality of the data and selected the first principal components to represent the true dimensionality of the data. Finally, we clustered the cells into 4 cell types via a K-nearest-neighbor approach with a resolution parameter of 0.6. Cell-type markers were detected by the Wilcoxon rank sum test. Cell cycle assignment was predicted by a machine learning–based approach, implemented in the Bioconductor package scran cyclone.^41^

### Isolation of DC fragments using a Complex Object Parametric Analyzer and Sorter (COPAS) large particle flow cytometer

COPAS sorting was performed as described earlier.^42^ In brief, Kl-KO^DC_Tomato^ and Control^Tomato^ mice were perfused via the left heart ventricle with 10 ml of ice-cold PBS, followed by 10 ml of digestion buffer (collagenase type 1, 1 mg/ml, Worthington; 0.1 mg/ml DNase, Sigma-Aldrich; 1 mg/ml hyaluronidase, Sigma-Aldrich; prepared in ice-cold KREBS buffer: NaCl 145 mM, *N*-2-hydroxyethylpiperazine-*N*′-2-ethanesulfonic acid 10 mM, KCl 5 mM, NaH_2_PO_4_ 1 mM, CaCl_2_ 2.5 mM, MgSO_4_ 1.8 mM, glucose 5 mM, pH 7.3). Kidneys were placed in ice-cold KREBS buffer and minced into small pieces. These pieces were then further digested in the digestion buffer (as described above) for 17 minutes at 37 °C. Digestion was followed by sieving of the tubular fluid through several different sieves, starting with a 250-μm and then 212-μm nylon sieve. The final step was the filtration of the sieved tubular fluid through a 100-μm and finally 40-μm cell strainer (Becton Dickinson Labware). The tubules that were retained by the 40-μm cell strainer were then dissolved in 50 ml of ice-cold KREBS buffer. Sorting was performed by using a large particle flow cytometer (BioSorter; Union Biometrica) with a 250-μm-diameter flow cell. The biosorter’s settings were as follows: extinction detector, 0.311 mW; pressure on the sample cup, 8.5 psi; pressure on the diverter, 0.9 psi; sheath flow rate, 55%; laser, 561 nm at 50 mW; and photomultiplier tubes: green 350 V, yellow 400 V, and red 650 V. The speed of the mixer was 40%. Per 2 ml Eppendorf tube, 1000 fluorescent DCs diluted in ice-cold KREBS buffer were collected and subsequently centrifuged at 800 g for 4 minutes. The supernatant KREBS buffer was removed, and the remaining pellet of DCs was resuspended in either RNA lysis buffer for RNA isolation or 50 μl 2× Laemmli buffer for Western blot analysis. Resuspended samples were then stored at −80 °C until further processing. In addition, some pellets were simply frozen down at −80 °C without resuspension.

### Bulk RNA-sequencing of DC cells

COPAS-sorted DC fragments from both Kl-KO^DC_Tomato^ and Control^Tomato^ mice were processed using a NucleoSpin kit to extract RNA as explained earlier. Subsequently, bulk RNA-seq analysis was performed commercially by Novo-gene (UK) Company Limited, as described earlier.^35,36^ In brief, amplified cDNA samples underwent rigorous quality control checks. The RNA samples were used for library preparation using the NEBNext Ultra RNA Library Prep Kit for Illumina, with the incorporation of indices for multiplexing purposes. Briefly, mRNA was purified from total RNA using poly-T oligo–attached magnetic beads. After fragmentation, the first-strand cDNA was synthesized using primers, succeeded by second-strand cDNA synthesis. The library was prepared after subsequent steps involving end repair, A-tailing, adapter ligation, and size selection. After amplification, the insert size of the library was confirmed using an Agilent 2100, and quantification was performed using quantitative PCR. The libraries were then sequenced on the Illumina NovaSeq 6000 S4 flow cell with PE150 sequencing, guided by the results from library quality control and anticipated data volume.

In the bulk RNA-seq data analysis, we included 8 datasets, with 4 animals per group. Fold changes were determined by dividing the arithmetic mean of the normalized read counts by the number of replicates. The statistical significance of the RNA-seq data was assessed with a P value threshold of <0.05. The heatmap of differentially expressed genes was generated using the R package ComplexHeatmap (version 2.4.3). The filling set of genes shows differential expression between the groups (*P* < 0.05) for both control and knockout mice. The input expression matrix was normalized by row (gene) through the computation of *z* scores. Both experimental groups (columns) and gene expression profiles (rows) were subjected to clustering using the Euclidean distance and the hierarchical clustering algorithm.

### Statistics

All the values are expressed as arithmetic means ± SEM, where n represents the number of animals. An unpaired Student *t* test, 1-way or 2-way analysis of variance was used for comparisons between the groups using GraphPad Prism (version 9.2.0). In cases in which the *P* value is not mentioned, the following applies: ns (not significant) *P* > 0.05, *P ≥ 0.05, **P < 0.01, and ***P < 0.001.

## RESULTS

### Klotho is enriched in DCT2/CNT with low expression in DCT1

The DCT is divided into an early portion (DCT1) and a late portion (DCT2), which ends in the CNT. Both DCT1 and DCT2 express the NCC whose abundance decreases along the DCT2 toward the CNT. The Ca^2+^ channel TRPV5 is present in the DCT2 and CNT. Recent scRNA-seq data suggest that *Kl* transcripts are expressed in both DCT and CNT segments (DCT and CNT together form the DC).^26,43^ Mice with a tamoxifen-inducible expression of the Cre-recombinase in the 3′ -untranslated region of the DCT-specific Ncc gene or the 3′ -untranslated region of the DCT2/CNT-specific *Trpv5* gene were crossed with Ai14 (Tomato) reporter mice to isolate fluorescent DC cells (Figure 1a). Earlier, similar genetic modifications in Ncc-Cre mice were generated and described previously,^28^ whereas the Trpv5-Cre mouse line is novel and was generated particularly for this project. Expression of the Cre-recombinase in the 3′ -untranslated region of the *Trpv5* gene slightly reduced TRPV5 expression in homozygous mice but not in heterozygous mice. Moreover, there was no effect on Klotho expression and all tested blood parameters (Supplementary Figure S1). Notably, Cre staining in the kidneys of tamoxifen-treated Trpv5^Cre/+^ mice is exclusively observed in the DCT2 and CNT (Supplementary Figure S2). Furthermore, detailed histological analysis of the Trpv5^CreERT2/0^Ai14^fl/+^ mouse line showed that cre-mediated recombination and hence tdTomato protein occurred exclusively in the kidney (Supplementary Figure S3).

We then conducted scRNA-seq on the isolated DC cells. Figure 1b indicates the gradual reduction of Ncc from the DCT to CNT, validating our approach. *Kl* transcripts correlated positively with DCT2/CNT markers (*Calb1, S100g*) and negatively with DCT1 markers (*Pvalb, Wfdc15b*), indicating that *Kl* is enriched in DCT2/CNT (Figure 1c). This was further supported by a positive correlation of *Kl* with *Trpv5* but not with Ncc expression levels (Figure 1d). Additional scRNA-seq analysis supporting cluster identification and gene expression gradients are shown in Supplementary Figures S4-S7 and Supplementary Tables S5 and S6. Immunofluorescent analysis confirmed prominent expression of Klotho in DCT2/CNT (Figure 1e; Supplementary Figure S8).

### Generation of a novel DC-specific Klotho knockout mouse model

**Figure 2 ∣:**
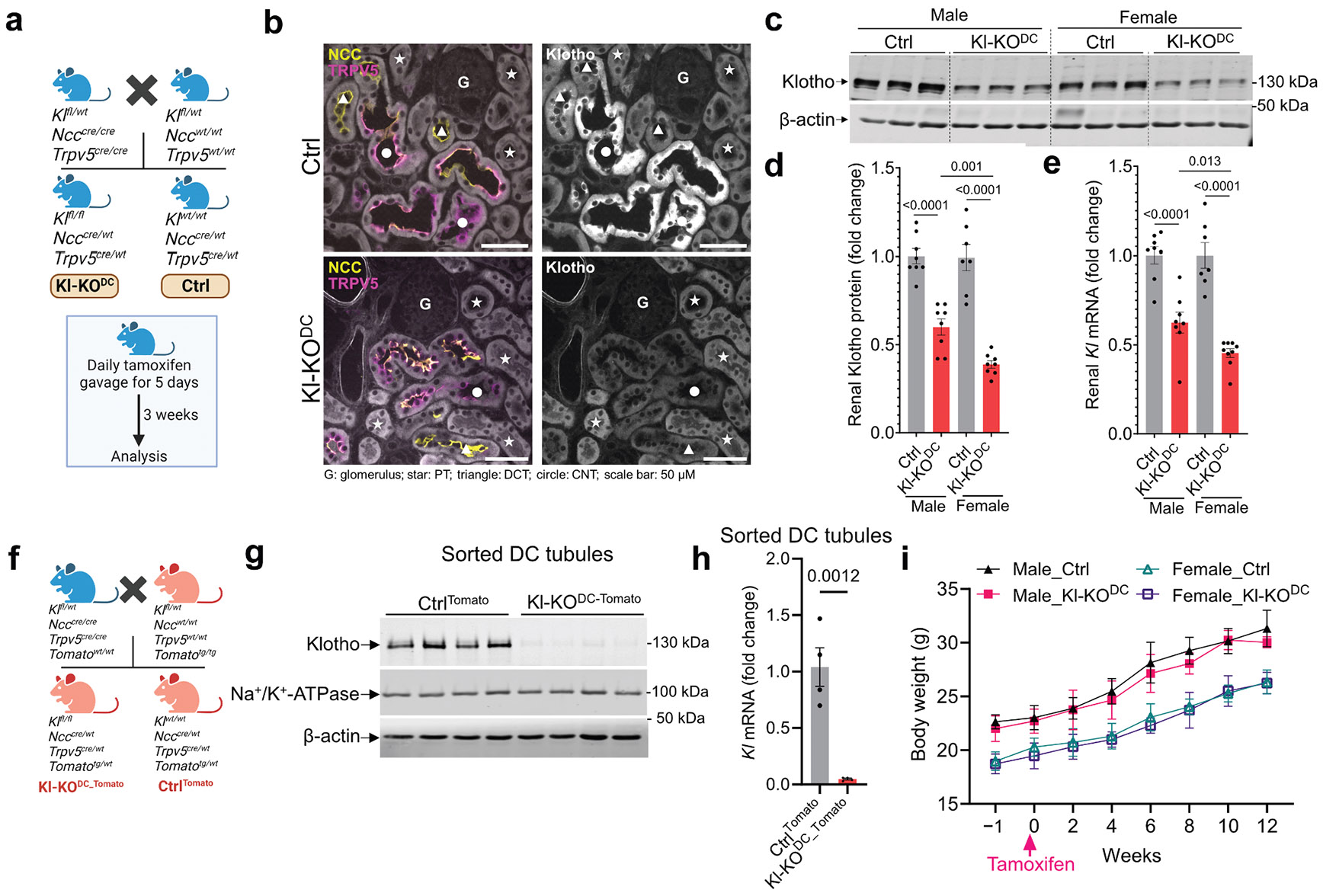
Generation of a novel distal convolution (DC)–specific Klotho knockout mouse model. (**a**) Mouse breeding strategies used to generate Kl-KO^DC^ and corresponding control mice. The mice received tamoxifen through 5 daily oral gavages and were analyzed 3 weeks after the last tamoxifen gavage. (**b**) Immunofluorescence analysis of consecutive kidney sections from control and Kl-KO^DC^ mice showing Na^+^ /Cl^−^ cotransporter (NCC) (yellow), transient receptor potential cation channel subfamily V member 5 (TRPV5) (magenta), and Klotho (bright) expression pattern. Note the undetectable Klotho in the DC of Kl-KO^DC^ mice, whereas Klotho in the PT remains unchanged. (**c**) Representative immunoblots of Klotho and β-actin in total kidney lysates from Kl-KO^DC^ and corresponding control mice. (**d**) Densitometric analysis of Klotho normalized to β-actin (values are mean ± SEM; n = 7–8 mice per group). (**e**) *Kl* mRNA levels quantified by quantitative reverse transcription polymerase chain reaction (qRT-PCR) in whole kidneys from Kl-KO^DC^ and corresponding control mice (n = 7–9 mice per group). (**f**) Mouse breeding strategies used to generate Kl-KO^DC_Tomato^ and Ctrl^Tomato^ mice. (**g**) Original immunoblot of Klotho, β-actin, and Na^+^ /K^+^ /ATPase in the DC of Kl-KO^DC_Tomato^ and corresponding control mice. β-Actin and Na^+^ /K^+^ /ATPase were used to demonstrate equal loading. (**h**) *Kl* mRNA levels in the DC of Kl-KO^DC_Tomato^ and corresponding control mice as measured by qRT-PCR (values are mean ± SEM; n = 4 per group). (**i**) The body weight progression in Kl-KO^DC^ and corresponding control mice up to 12 weeks after tamoxifen induction (values are mean ± SEM; n = 5–7 mice per group). ATPase, adenosine triphosphatase; CNT, connecting tubule; DCT, distal convoluted tubule; PT, proximal tubule. To optimize viewing of this image, please see the online version of this article at www.kidney-international.org.

The *Kl^fl/fl^* mice were custom-made as described in the Methods section. The insertion of loxP sequences in the *Kl* gene did not affect the renal expression of Klotho at protein or mRNA levels (Supplementary Figure S9). The *Kl^fl/fl^* mice were crossbred with *Ncc^cre^* and *Trpv5^cre^* to generate novel inducible DC-specific Klotho knockout models (*Kl^fl/fl^Ncc^cre^ Trpv5^cre^*, herein Kl-KO^DC^) as shown in Figure 2a. The Kl-KO^DC^ mice were compared with Ctrl mice (*Kl^+/+^ Ncc^cre^ Trpv5^cre^*).

Three weeks after tamoxifen induction, efficient deletion of Klotho specifically in the DC was confirmed via immunofluorescence and RNAscope on kidney sections (Figure 2b; Supplementary Figure S10). Anti-NCC and anti-TRPV5 stains were used to identify DCT and DCT2/CNT, respectively. Klotho protein and mRNA levels in PT were unchanged. A 40%–50% reduction in Klotho protein and mRNA levels in total kidney was observed, indicating that the remaining Klotho was derived from the PT (Figure 2c-e). Although Klotho expression in individual PT cells is modest, these cells vastly outnumber DC cells in the whole kidney.^44^ Thus, because of its larger cell population, PT contributes substantially to the whole kidney Klotho protein and mRNA abundance. *Kl* mRNA levels in the parathyroid and brain of Kl-KO^DC^ mice remained unchanged (Supplementary Figure S11). In addition, as shown in Figure 2f, a Kl-KO^DC_Tomato^ mouse model was generated for automated sorting of fluorescent DC tubules. As shown in Figure 2g and h, DC segments isolated from Kl-KO^DC_Tomato^ mice exhibited negligible Klotho protein and mRNA expression, further confirming efficient Klotho deletion. Interestingly, Kl-KO^DC^ mice displayed no overt clinical phenotype and maintained stable body weight up to 12 weeks after induction (Figure 2i). No significant changes were observed in tested blood parameters (Supplementary Table S7).

### Klotho deficiency in DC does not affect Pi homeostasis, but pan-tubular Klotho deficiency rapidly causes hyperphosphatemia and FGF23 resistance

**Figure 3 ∣:**
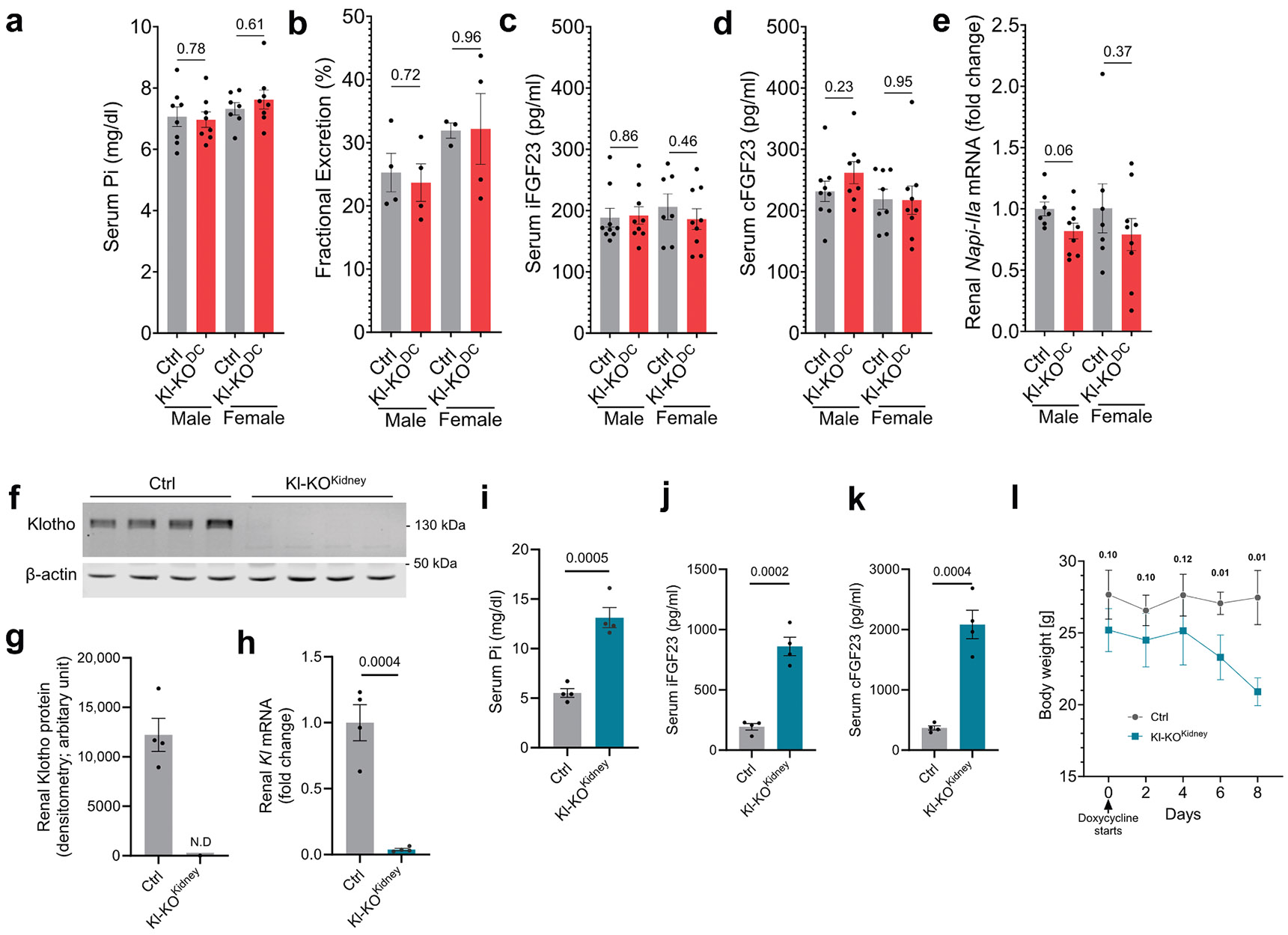
Klotho deficiency in distal convolution (DC) does not affect phosphate (Pi) homeostasis, but pan-tubular Klotho deficiency rapidly causes hyperphosphatemia and fibroblast growth factor-23 (FGF23) resistance. (**a**) Serum Pi levels in Kl-KO^DC^ and corresponding control mice (values are mean ± SEM; n = 7–8 each group). (**b**) Fractional excretion (FE) of Pi was measured in 24-hour urine from Kl-KO^DC^ and corresponding control mice (values are mean ± SEM; n = 3–4 mice per group). Serum (**c**) intact fibroblast growth factor-23 (iFGF23) and (**d**) c-termFGF23 levels in Kl-KO^DC^ and corresponding control mice (n = 7–9 mice per group). (**e**) *NaPi-IIa* mRNA levels quantified by quantitative reverse transcription polymerase chain reaction (qRT-PCR) in kidneys from Kl-KO^DC^ and corresponding control mice (values are mean ± SEM; n = 7–9 mice per group). (**f**) Original immunoblot of Klotho and β-actin in the total kidney lysate of Kl-KO^Kidney^ and corresponding control mice. (**g**) Densitometric analysis of Klotho normalized to β-actin (values are mean ± SEM; n = 4 mice per group; ND: not detected). (**h**) *Kl* mRNA levels in the total kidney of Kl-KO^Kidney^ and corresponding control mice as measured by qRT-PCR (values are mean ± SEM; n = 4 per group). (**i**) Serum Pi levels in Kl-KO^Kidney^ and corresponding control mice (values are mean ± SEM; n = 4 each group). Serum (**j**) iFGF23 and (**k**) c-termFGF23 levels in Kl-KO^Kidney^ and corresponding control mice (values are mean ± SEM; n = 4 mice per group). (**l**) The body weight progression in Kl-KO^Kidney^ and corresponding control mice up to 8 days after starting doxycycline induction (values are mean ± SEM; n = 4 mice per group).

We found no change in Pi levels in serum and 24-hour urine (Figure 3a and b) in Kl-KO^DC^ mice compared with control mice. Intact and C-terminal FGF23 levels in serum, measured by ELISA, were also not significantly altered in Kl-KO^DC^ mice (Figure 3c and d). Finally, *NaPi-IIa* expression levels quantified by qRT-PCR (Figure 3e) and immunoblot (Supplementary Figure S12) in whole kidneys were not statistically different between Kl-KO^DC^ and control mice. One limitation is that NaPi-IIa abundance was assessed in whole-kidney lysates rather than in brush border-membrane vesicle preparations, which more directly reflect apical NaPi-IIa abundance and activity.

Next, we generated a novel doxycycline-inducible, kidney-specific (pan-tubular) Klotho knockout mouse model (Kl-KO^Kidney^) by crossing Kl^fl/fl^ mice with previously described Pax8rtTA-LC1 mice.^30^ In induced Kl-KO^Kidney^ mice, Klotho protein and *Kl* mRNA levels were absent or nearly undetectable in the total kidney lysates of Kl-KO^Kidney^ mice (Figure 3f-h). Notably, serum Pi levels (Figure 3i) were significantly elevated in the Kl-KO^Kidney^ mice compared with control mice. The concentrations of Ca^2+^ and K^+^ in the blood were also increased, whereas the other tested blood parameters remained unchanged (Supplementary Table S8). Moreover, both serum intact and C-terminal FGF23 were markedly increased in Kl-KO^Kidney^ mice. Furthermore, Kl-KO^Kidney^ mice exhibited a progressive decline in body weight starting at day 6 after initiating doxycycline-induced recombination (Figure 3l).

### DC is the primary source of urinary sKlotho, whereas PT is a likely source of serum sKlotho in mice

**Figure 4 ∣:**
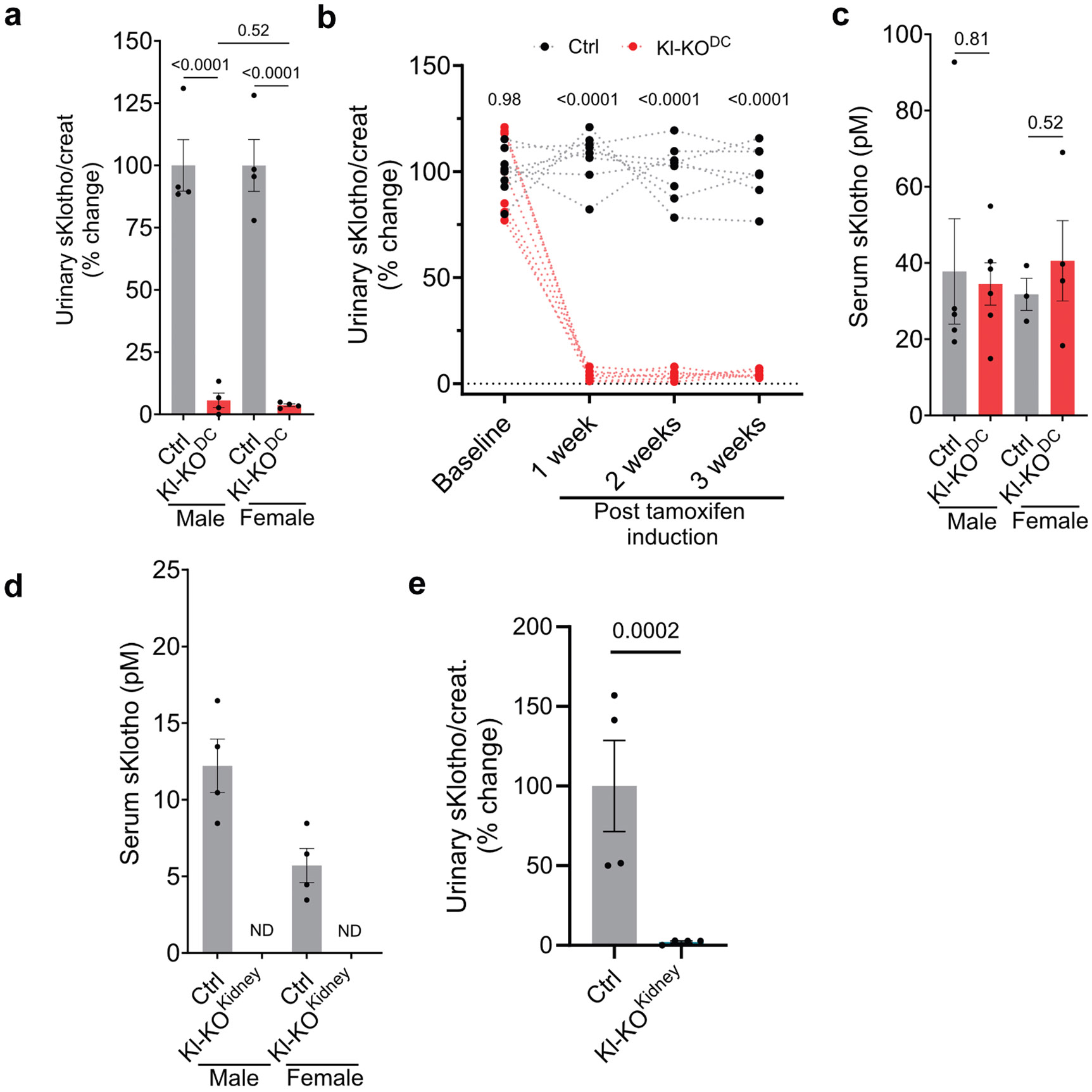
Distal convolution (DC) is the primary source of urinary soluble Klotho (sKlotho), whereas the proximal tubule (PT) is a likely source of serum sKlotho in mice. (**a**) sKlotho levels in 24-hour urine, normalized to creatinine in Kl-KO^DC^ and corresponding control mice (values are mean ± SEM; n = 4 per group). (**b**) Urinary sKlotho levels normalized to creatinine in baseline spot urine (immediately before tamoxifen induction) and spot urine samples collected weekly after oral tamoxifen gavage for 5 consecutive days (values are mean ± SEM; n = 5–7 per group). (**c**) Serum sKlotho levels measured by immunoprecipitation-immunoblot (IP/IB) in Kl-KO^DC^ and corresponding control mice (values are mean ± SEM; n = 3–6 per group). (**d**) Serum sKlotho levels measured by IP/IB in Kl-KO^Kidney^ and corresponding control mice (values are mean ± SEM; n = 3–4 per group). (**e**) Urinary sKlotho levels normalized to creatinine in spot urine samples of Kl-KO^Kidney^ and corresponding control mice (values are mean ± SEM; male, n = 4 per group).

We measured the levels of sKlotho in both urine and serum from control and Kl-KO^DC^ mice. Although commercial ELISA or immunoblot can quantify urinary sKlotho, serum sKlotho could be detected accurately in mice only with an immunoprecipitation-immunoblot assay as demonstrated in previous studies.^33,45^ We validated the urinary sKlotho detection by ELISA by spiking recombinant sKlotho into mouse urine (Supplementary Figure S13A). Using metabolic cages with a urine-cooling apparatus, we collected 24-hour urine samples at 4 °C. Remarkably, sKlotho was nearly undetectable in 24-hour urine samples from Kl-KO^DC^ mice as shown by ELISA (Figure 4a) as well as by immunoblotting (Supplementary Figure S13B). To track the decline of urinary sKlotho in mice with a DC-specific deletion of Klotho, we analyzed spot urine samples before and several days after tamoxifen induction. Within 1 week, sKlotho disappeared almost completely from the urine (Figure 4b). Surprisingly, the serum sKlotho levels, measured by the immunoprecipitation-immunoblot method, remained unchanged in Kl-KO^DC^ mice (Figure 4c). In contrast, serum sKlotho was undetectable in Kl-KO^Kidney^ mice, as determined by the same immunoprecipitation-immunoblot assay (Figure 4d), confirming that loss of Klotho throughout the kidney tubules, including the PT, abolishes circulating sKlotho. Urinary sKlotho levels, as measured by ELISA, were undetectable in Kl-KO^Kidney^ mice (Figure 4e).

### Transcriptomic analysis of Klotho-deficient DC reveals inactivation of MAPK signaling

**Figure 5 ∣:**
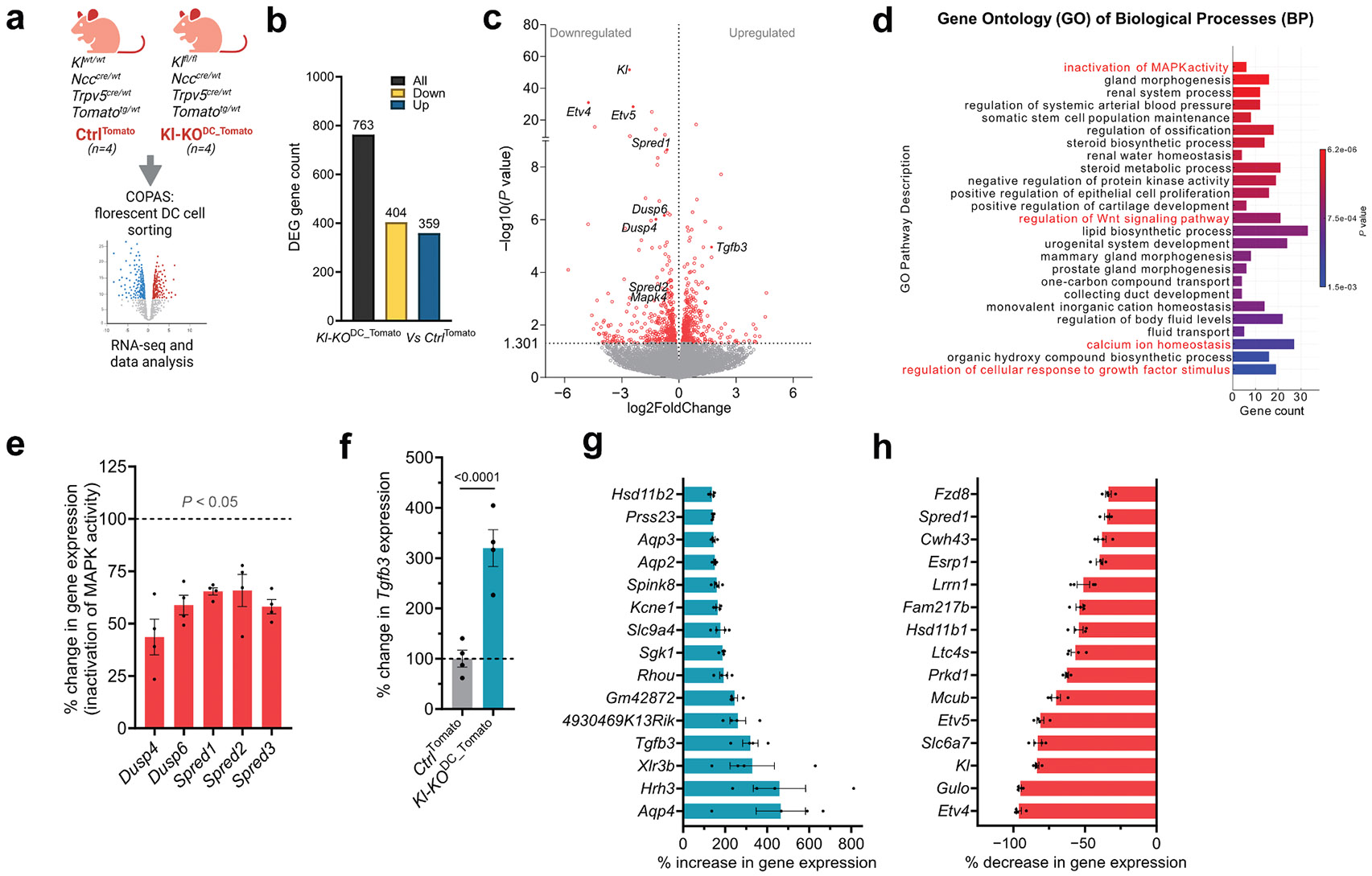
Transcriptomic analysis of Klotho-deficient distal convolution (DC) reveals inactivation of mitogen-activated protein kinase (MAPK) signaling due to impaired fibroblast growth factor-23 (FGF23)–FGF receptor (FGFR)–Klotho binding. (**a**) DC cells were sorted from Kl-KO^DC_Tomato^ and Ctrl^Tomato^ mice 3 weeks after tamoxifen induction (n = 4 per group). RNA-sequencing analysis was performed on sorted DC cells from these mice. (**b**) Differentially expressed gene (DEG) counts in Kl-KO^DC_Tomato^ compared with Ctrl^Tomato^ mice. (**c**) Volcano plot showing upregulated/downregulated genes in DC from Kl-KO^DC_Tomato^ compared with Ctrl^Tomato^ mice. (**d**) Top Gene Ontology (GO) pathways implicated in the analysis of biological processes (BP) of all the DEGs (*P <* 0.05). (**e**) Bar graphs depicting statistically significant downregulated genes implicated in the *inactivation of MAPK activity* (values are mean ± SEM; n = 4 per group; *P <* 0.05). (**f**) Bar graph showing *Tgfb3* expression in the DC of Kl-KO^DC_Tomato^ mice (values are mean ± SEM; n = 4 per group). Top 15 (**g**) upregulated and (**h**) downregulated genes in the DC of Kl-KO^DC_Tomato^ mice compared with Ctrl^Tomato^ mice (values are mean ± SEM; n = 4 per group; *P <* 0.05). COPAS, Complex Object Parametric Analyzer and Sorter.

We found that Klotho protein and mRNA were almost undetectable in COPAS-sorted DC of Kl-KO^DC_Tomato^ mice (Figure 2c). The deletion of Klotho from DC cells may impair FGF23-FGFR-Klotho assembly, thus affecting the transcriptome. To assess the effect of deletion of Klotho on the transcriptome of DC cells, we performed bulk RNA-seq on isolated DC fragments from Ctrl^Tomato^ and Kl-KO^DC_Tomato^ mice (Figure 5a). We identified 763 differentially expressed genes: 404 upregulated and 359 downregulated (Figure 5b and volcano plot, Figure 5c). A heatmap of the differentially expressed genes is available in Supplementary Figure S14. Gene Ontology analysis of biological processes revealed the top pathways, with “inactivation of MAPK activity” significantly altered (*P* < 0.05) (Figure 5d). The other impaired Gene Ontology pathways were “regulation of Wnt signaling” and “calcium ion homeostasis.” The complete list of affected pathways is provided in the Supplementary Dataset. Genes (*Dusp4/6, Spred1/2/3*) involved in the MAPK signaling pathway are depicted in Figure 5e. *Tgfb3* (transforming growth factor beta 3), which is known to interact with MAPK,^46^ exhibited a 3-fold upregulation in the DC from Kl-KO^DC_Tomato^ mice (Figure 5g). The top 15 upregulated and downregulated genes in Klotho-deficient DC are depicted in Figure 5h and i, respectively. Notably, qRT-PCR analysis showed a 95% reduction in *Kl* mRNA levels in DC from Kl-KO^DC_Tomato^ mice (see Figure 2d), whereas RNA-seq revealed an 84% reduction, probably attributed to the lower sensitivity of RNA-seq compared with qRT-PCR.

### Klotho in DC regulates TRPV5, renal Ca^2+^ reabsorption, and bone density

**Figure 6 ∣:**
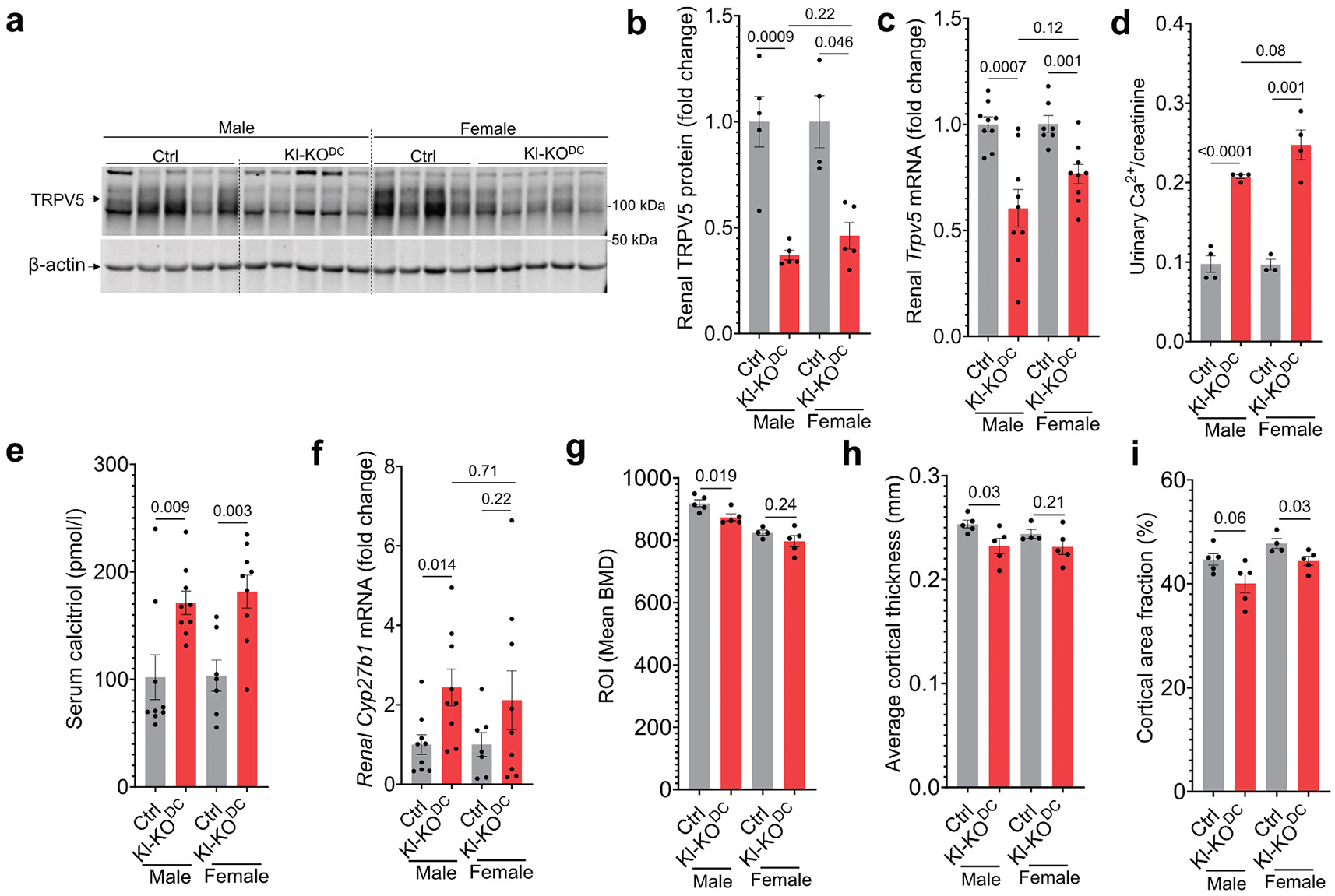
Klotho in distal convolution (DC) regulates Ca^2+^ reabsorption and bone remodeling. (**a**) Original immunoblot of transient receptor potential cation channel subfamily V member 5 (TRPV5) and β-actin in total kidney lysates from Kl-KO^DC^ and corresponding control mice. (**b**) Densitometric analysis of TRPV5 normalized to β-actin (values are mean ± SEM; n = 4–5 mice per group). (**c**) *Trpv5* mRNA levels quantified by quantitative reverse transcription polymerase chain reaction (qRT-PCR) in whole kidneys from Kl-KO^DC^ and corresponding control mice (values are mean ± SEM; n = 7–9 mice per group). (**d**) Urinary Ca^2+^ excretion measured in 24-hour urine, normalized to creatinine in Kl-KO^DC^ and corresponding control mice (values are mean ± SEM; n = 3–4 mice per group). (**e**) Serum calcitriol levels in Kl-KO^DC^ and corresponding control mice (values are mean ± SEM; n = 7–9 mice per group). (**f**) *Cyp27b1* mRNA levels quantified by qRT-PCR in kidneys from Kl-KO^DC^ and corresponding control mice (values are mean ± SEM; n = 7–9 mice per group). Bone density parameters, namely (**g**) mean bone mineral density (BMD), (**h**) average cortical thickness, and (**i**) cortical area fraction in the hindlegs of Kl-KO^DC^ and corresponding control mice (values are mean ± SEM; n = 4–5 mice per group). ROI, region of interest.

Both male and female Kl-KO^DC^ mice exhibited a statistically significant downregulation of TRPV5 protein and mRNA levels by immunoblotting and qRT-PCR in whole kidney (Figure 6a-c). *Calb1* (calbindin) mRNA was also reduced in male mice (Supplementary Figure S15). Kl-KO^DC^ mice showed significant hypercalciuria in 24-hour urine samples, with urinary Ca^2+^ levels normalized to creatinine twice as high as in control mice (Figure 6d). Plasma Ca^2+^ levels remained unchanged (Supplementary Table S3). Serum calcitriol levels were significantly higher in Kl-KO^DC^ mice (Figure 6e), whereas serum parathyroid hormone levels measured in male mice were unchanged between the groups (Supplementary Figure S16). *Cyp27b1* mRNA was statistically significantly higher in male Kl-KO^DC^ mice, with a similar trend in female mice (Figure 6f). Renal *Cyp24a1* mRNA levels were not significantly different between groups (Supplementary Figure S15). Micro–computed tomography analysis of femurs revealed statistically significant reductions or similar strong trends in bone remodeling parameters such as BMD, cortical thickness, and cortical area fraction in Kl-KO^DC^ mice (Figure 6g-i).

### Hypercalciuria in Kl-KO^DC^ mice is primarily due to the loss of Klotho in DCT2/CNT

**Figure 7 ∣:**
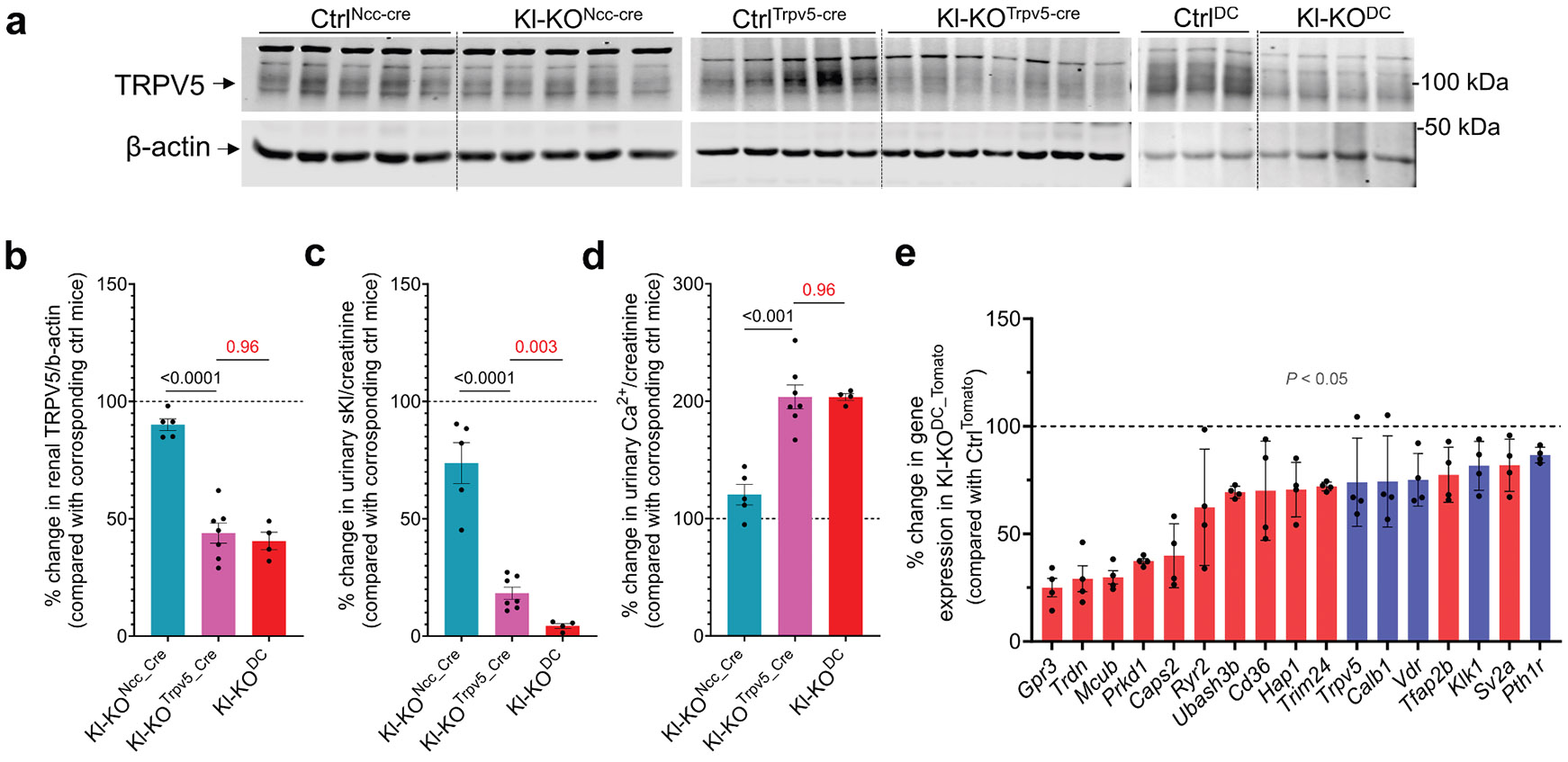
Hypercalciuria in Kl-KO^DC^ mice is primarily due to the loss of Klotho in distal convoluted tubule (DCT)2/connecting tubule (CNT), rather than in DCT1 or changes in urinary soluble Klotho (sKlotho) levels. (**a**) Original immunoblot of transient receptor potential cation channel subfamily V member 5 (TRPV5) and β-actin in total kidney lysates from Kl-KO^Ncc_Cre^, Kl-KO^Trpv5_Cre^, Kl-KO^DC^, and corresponding control mice. (**b**) Densitometric analysis of TRPV5 normalized to β-actin (values are mean ± SEM; n = 3–7 mice per group). (**c**) sKlotho levels in 24-hour urine, normalized to creatinine in Kl-KO^Ncc_Cre^, Kl-KO^Trpv5_Cre^, Kl-KO^DC^, and corresponding control mice (values are mean ± SEM; n = 3–7 mice per group). (**d**) Urinary Ca^2+^ excretion measured in 24-hour urine, normalized to creatinine in Kl-KO^Ncc_Cre^, Kl-KO^Trpv5_Cre^, Kl-KO^DC^, and corresponding control mice (values are mean ± SEM; n = 3–6 mice per group). (**e**) Extended transcriptomics analysis in distal convolution (DC) of Kl-KO^DC_Tomato^ compared with Ctrl^Tomato^. Bar graphs depicting statistically significant downregulated genes implicated in the Gene Ontology (GO) pathway of biological processes related to *calcium ion homeostasis.* The blue bars indicate known Ca^2+^ -regulating genes expressed in DCT2/CNT. The red bars indicate genes involved in Ca^2+^ transport but is not implicated in renal Ca^2+^ reabsorption (values are mean ± SEM; n = 4 mice per group; *P* < 0.05).

To investigate which portion of the DC is most crucial for Klotho-dependent regulation of Ca^2+^ reabsorption, we generated mouse models lacking Klotho exclusively in DCT cells (Kl-KO^Ncc-Cre^) and DCT2/CNT cells (Kl-KO^Trpv5-Cre^). We found a subtle reduction in renal TRPV5 expression in Kl-KO^Ncc-Cre^ compared with Kl-KO^Trpv5-Cre^ and Kl-KO^DC^ mice (Figure 7a and b; Supplementary Figure S17A). The reduction in TRPV5 protein was similar in Kl-KO^Trpv5-Cre^ and Kl-KO^DC^ mice (Figure 7a and b; Supplementary Figure S17A). Urinary sKlotho levels and Ca^2+^ excretion were assessed in 24-hour urine samples. Urinary sKlotho dropped by around 20% and 80% in Kl-KO^Ncc-Cre^ and Kl-KO^Trpv5-Cre^ mice, respectively (Supplementary Figure S17B), consistent with our scRNA-seq data. Around 95% drop in urinary sKlotho was observed in Kl-KO^DC^ compared with control mice (Figure 7c; Supplementary Figure S17B). The 24-hour urinary Ca^2+^ excretion was slightly higher in Kl-KO^Ncc-Cre^ mice than in control mice. Two-fold higher Ca^2+^ excretion was observed in both Kl-KO^Trpv5-Cre^ and Kl-KO^DC^ mice compared with controls, with no significant difference between Kl-KO^DC^ and Kl-KO^Trpv5-Cre^ mice (Figure 7d; Supplementary Figure S17C). Consistent with these physiological findings, transcriptomic analysis of DC cells from Kl-KO^DC_Tomato^ mice revealed significant enrichment of the Gene Ontology BP pathway “calcium ion homeostasis” (*P* = 0.001) with coordinated downregulation of canonical Ca^2+^ regulating genes such as *Trpv5, Calb1, Vdr, Klk1*, and *Pth1r* (Figure 7e; see Figure 5d). When compared with the scRNA-seq of DC, these transcripts showed a strong positive correlation with *Kl* (DCT2/CNT marker) but a negative or no correlation with a DCT1 marker *Pvalb* (Supplementary Figure S18).

It has been proposed that FGF23-Klotho signaling regulates NCC expression and sodium handling in the DCT, and that reduced NCC activity, natriuresis, and volume contraction can enhance Ca^2+^ reabsorption in upstream nephron segments.^47,48^ To evaluate the contribution of NCC to the altered calciuria observed in Kl-KO^DC^ mice, we quantified NCC and phosphorylated NCC abundance in kidney lysates from Kl-KO^DC^, Kl-KO^Ncc-Cre^, and Kl-KO^Trpv5-Cre^ mice. Both NCC and phosphorylated NCC were significantly reduced only in Kl-KO^DC^ and Kl-KO^Ncc-Cre^ mice (Supplementary Figure S19). To test if decreased NCC activity promotes hypocalciuria in a Klotho-independent manner, we measured urinary Ca^2+^ excretion in NCC-KO mice (NCC^−/−^) (Supplementary Figure S20A and B). As expected, we observed significant hypocalciuria in NCC^−/−^ without any change in Klotho expression in comparison with NCC^+/+^ (Supplementary Figure S20C and D).

## DISCUSSION

The main findings of our study are as follows: (i) Within DC, Klotho is enriched in DCT2/CNT with lower expression in DCT1. (ii) DC-derived Klotho does not contribute to renal Pi homeostasis. (iii) Klotho in DC contributes to urinary but not circulatory sKlotho levels. (iv) Klotho in PT maintains Pi homeostasis and likely regulates circulating sKlotho levels. (v) MAPK signaling is suppressed in Klotho-deficient DC. (vi) DC-derived Klotho in DCT2/CNT cells is crucial for renal Ca^2+^ homeostasis.

Using scRNA-seq on isolated DC cells, we found that *Kl* transcripts are more abundant in DCT2/CNT than in DCT1. Immunofluorescence of kidneys with nephron segment-specific markers confirmed this pattern at the protein level. Multiple scRNA-seq resources indicate that in the mouse kidney Klotho is enriched in DCT2/CNT, whereas human and rat datasets show comparatively higher expression within the DCT (Humphreys Lab SingleCell Atlas; Nephron RNA-seq database). In contrast, Jung *et al*.^49^ recently demonstrated that, at the protein level, Klotho is highly expressed in the late DCT and CNT in both mice and humans. These differences likely arise from methodological issues, such as the selection of marker genes and clustering strategies in scRNA-seq analyses. Taken together, these data highlight that interspecies and methodological differences must be considered, and direct extrapolation to human physiology should be made with caution.

The lack of hyperphosphatemia and FGF23 resistance in Kl-KO^DC^ mice is likely explained by the fact that expression of Klotho in the PT remained intact, which contributes to Pi and vitamin D homeostasis.^8,10,50^ The present findings do not support the long-held purported hypothesis that the DC secretes sKlotho as a paracrine factor affecting the PT to regulate Pi homeostasis.^3,23,24,51^ Our findings are in line with a recent study suggesting the central role of Klotho in PT in Pi homeostasis.^13^ The authors found that lack of Klotho in PT in mice led to elevated plasma Pi, FGF23, and calcitriol levels, leading to ectopic calcification and aging-like phenotypes, similar to global Klotho knockout. However, our results are not in agreement with another study showing that Klotho deficiency in PT caused mild or no hyperphosphatemia with modest changes in calcitriol and FGF23 levels.^24^ The reasons for this discrepancy are unclear but can possibly be due to the cell-type differences in Cre expression, Cre expression efficiency, and the feasibility of recombination based on the lox P insertion site structure in the genome. Moreover, the Klotho^flox/flox^ mouse strain used in previous studies differed from ours.

On the other hand, our novel inducible Kl-KO^Kidney^ mouse model exhibited rapid hyperphosphatemia, elevated FGF23 levels, and weight loss similar to the previously reported mice with a global knockout of Klotho^1^ or a constitutive deletion of Klotho specifically in either the kidney^7^ or the PT.^13^ Thus, Klotho deletion that includes the PT invariably causes a severe phenotype with hyperphosphatemia and high FGF23,^1,7^ whereas a DC-specific deletion of Klotho, as achieved in our Kl-KO^DC^ mice, induces hypercalciuria and lower bone density, but without hyperphosphatemia and any signs of general illness. The previous findings from animal experiments suggest that either reducing renal Pi reabsorption^52,53^ or decreasing calcitriol synthesis^47,54-56^ rescues the aging-like phenotypes in global Klotho knockout mice. Notably, both Pi reabsorption and calcitriol synthesis are PT functions.^52-54,56^

One key finding of our study is that DC-derived Klotho is mainly secreted into urine but not into the circulation. The unchanged serum sKlotho levels in Kl-KO^DC^ mice were unexpected but was unequivocal. In an earlier study, kidney-specific Klotho knockout mice exhibited approximately 80% reduction in serum sKlotho; however, urinary sKlotho was not assessed.^7^ Another study highlighted the kidney as the primary source of circulatory sKlotho, noting higher sKlotho levels in the suprarenal compared to the infrarenal inferior vena cava.^45^ Mice with PT-specific Klotho deletion did not exhibit reduced serum sKlotho levels.^24^ As such, the source tissue contributing to systemic serum sKlotho levels was not resolved. The Kl-KO^DC^ mice exhibited reduced urinary sKlotho levels only, whereas Kl-KO^Kidney^ mice showed loss of both serum and urinary sKlotho levels, supporting that the PT is a likely source of circulating sKlotho levels. However, this requires further experimental validation. Primary cultures of PT and DCT/CNT cells grown as polarized epithelia on permeable supports (e.g., Transwell) may allow to address the differential polarization of Klotho secretion assuming differentiation and Klotho expression persist in Transwell culture. However, such studies would be technically very challenging (if feasible at all). There are no robust primary cultures, or cell lines, that (i) retain native Klotho expression for PT and DC cells and (ii) grow reliably on Transwell filters to permit clean apical versus basolateral secretion assays. Therefore, we refrained from trying this approach. Previous *in vitro* studies using Klotho transfected MDCK cells demonstrated that Klotho is predominantly secreted apically.^57^ Future investigations will be required to elucidate the mechanisms underlying apical versus basolateral secretion of Klotho and to determine whether this process is differentially regulated between proximal and distal nephron segments.

Our unbiased and comprehensive RNA-seq of isolated DC suggests that Klotho deficiency in the DC reduces MAPK signaling. Several late-ERK targets such as *Dusp4/6, Spred1/2/3*, and *Etv4/5* were significantly downregulated in the DC of Kl-KO^DC_Tomato^ mice. This aligns with prior *in vitro* studies supporting the interaction between FGFR and Klotho, enabling FGF23 to activate MAPK and pERK1/2 signaling.^36,58-62^ Few studies also have shown pERK1/2 activation in the murine DC on exogenous FGF23 treatment.^51,63^ However, early-ERK targets such as *Egr1, Fos*, and Jun remained unchanged in the DC of Kl-KO^DC_Tomato^ mice, likely because they are primarily altered following acute FGF23 treatment.^36,64,65^ Our findings suggest that the FGF23-Klotho axis exhibits different temporal ERK targets in vivo. This is consistent with a recent elegant report from the White lab using scRNA-seq of the kidney after acute FGF23 treatment,^66^ as well as with our ongoing work examining acute and chronic FGF23 signaling dynamics.^36^ The Gene Ontology pathway analysis in our study also revealed suppressed Wnt signaling in the DC of Kl-KO^DC_Tomato^ mice, consistent with previous study demonstrating crosstalk between MAPK and Wnt signaling mediated by the FGF23-Klotho axis.^63^

In Kl-KO^DC^ mice, hypercalciuria is the primary disturbance, while serum Ca^2+^ levels remain unchanged. The FGF23-FGFR-Klotho complex in PT suppresses *Cyp27b1* expression, an enzyme necessary for calcitriol formation. Thus, global-Klotho knockout mice exhibit excessive serum calcitriol levels, increasing bone resorption, intestinal absorption, elevated serum Ca^2+^ levels, and increased renal Ca^2+^ excretion.^54^ However, Klotho in PT is intact in Kl-KO^DC^ mice, ruling out the *Cyp27b1* pathway in causing the hypercalciuria. In line with previous study,^55^ impaired FGF23-FGFR-Klotho binding and disrupted MAPK signaling in DC likely inhibited TRPV5 and Ca^2+^ transport in DC. The subsequent hypercalciuria may have led to significantly reduced BMD in Kl-KO^DC^ mice. The elevated *Cyp27b1* mRNA and serum calcitriol in Kl-KO^DC^ mice are modest compared with PT-specific or global-Klotho knockout mice. This rise is most likely a compensatory response to maintain serum Ca^2+^ levels in the presence of the profound hypercalciuria in Kl-KO^DC^ mice. Reduced renal NCC expression in these mice aligns with crosstalk between the FGF23–Klotho axis and sodium handling but is unlikely to cause hypercalciuria.^47,48,67-69^

**Figure 8 ∣:**
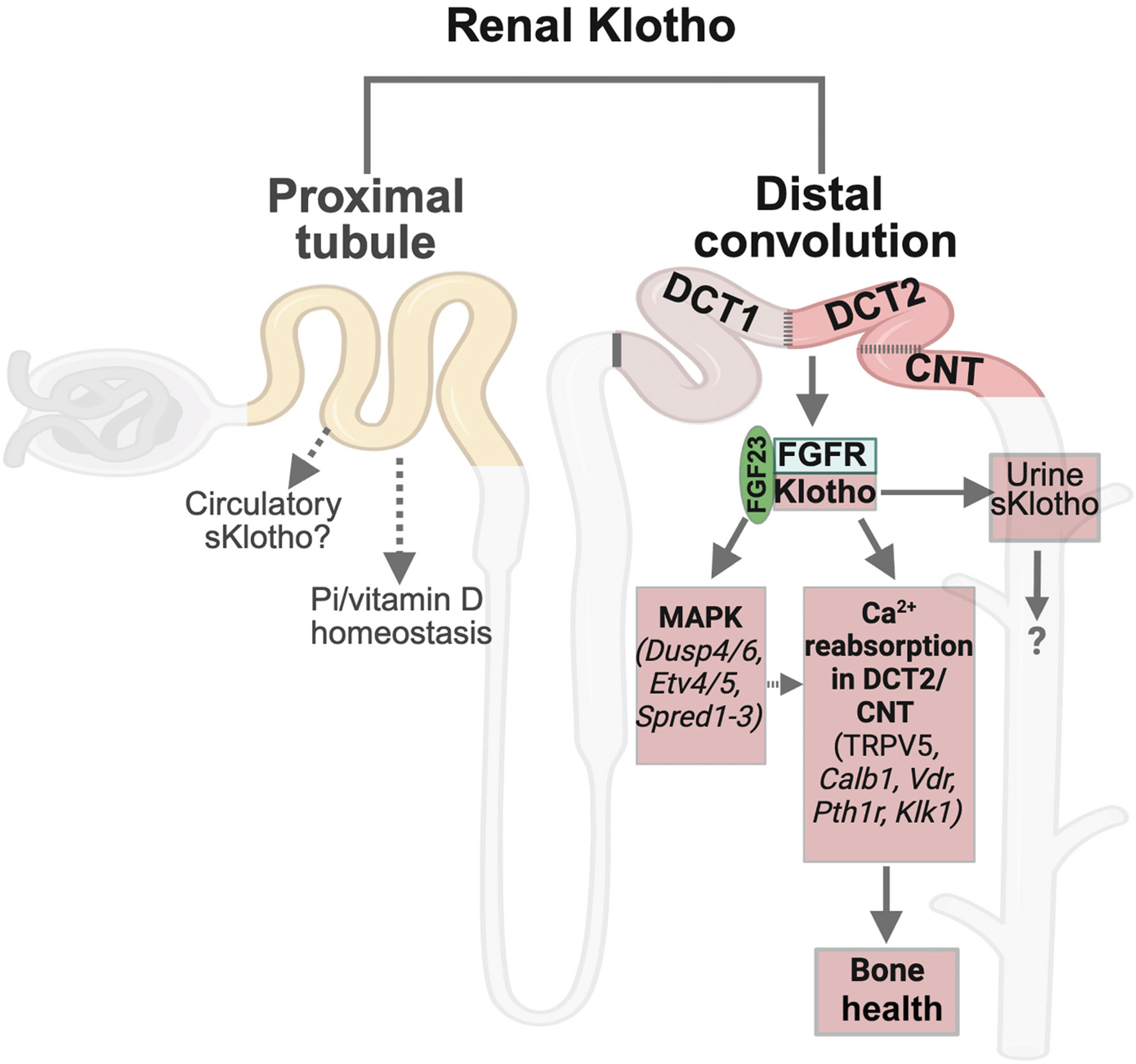
Proposed model for the role of Klotho in the kidney. Proximal tubule (PT) nephron (transparent yellow) segments have modest expression of Klotho. We found that within the distal convolution (DC), distal convoluted tubule (DCT)1 (in pink) has low expression of Klotho, whereas DCT2/connecting tubule (CNT) (in red) are highly enriched in Klotho. Urinary soluble Klotho (sKlotho) is primarily derived from these nephron segments, while PT likely contributes to circulatory sKlotho. The fibroblast growth factor-23 (FGF23)–FGF receptor (FGFR)–Klotho complex in DCT2/CNT regulates mitogen-activated protein kinase (MAPK) signaling and Ca^2+^ transporters. It is currently unclear whether and how MAPK directly regulates Ca^2+^ transporters. The FGF23-FGFR-Klotho–mediated renal Ca^2+^ reabsorption is crucial for maintaining bone health. On the other hand, lack of a phenotype in phosphate (Pi) homeostasis in Kl-KO^DC^ mice suggests that Klotho has 2 distinct roles in the kidney: in the PT for Pi/vitamin D balance and in the DC for regulating urinary sKlotho levels and Ca^2+^ reabsorption.

DCT2 cells resemble CNT cells and differ from DCT1 cells by exhibiting higher expression levels of proteins involved in transcellular Ca^2+^ transport.^70,71^ Consistently, the present study confirms that DCT2/CNT strongly expresses Klotho as well as the transcripts involved in transcellular Ca^2+^ reabsorption (*Trpv5, Calb1, Klk1, Vdr*, and *Pth1r*) (Supplementary Figure S18). Notably, these transcripts were significantly downregulated in Kl-KO^DC^ mice. Although Klotho is known to regulate TRPV5 and calbindin D28k at the protein level, this is the first report showing that Klotho deletion impacts their mRNA expression and other genes (e.g., *Klk1, Vdr*, and *Pth1r*) previously linked to Ca^2+^ transport but not to Klotho signaling.^72^ Our findings partly align with an earlier report showing that FGF23 regulates Ca^2+^ reabsorption in the DC of mice with inactive vitamin D receptor,^55^ but it also highlights that vitamin D receptor is involved in this process.^73-75^ Although the underlying molecular mechanisms are unclear, our data indicate that the FGF23-FGFR-Klotho complex in DCT2/CNT is critical for the regulation of Ca^2+^ transport by regulating the expression of *Trpv5, Vdr, Pth1r, Calb1*, and *Klk1* (Figure 8). Evolutionarily, Klotho emerged in vertebrates with bony skeletons, coinciding with the appearance of distinct nephron segments in these species.^76^ Therefore, we propose that the critical role of Klotho in the DCT2/CNT is to regulate renal Ca^2+^ reabsorption and to maintain bone health (Figure 8).

Previous data showed that Klotho may also regulate ROMK.^18^ Although Klotho and ROMK are colocalized in DC,^26^ we found that a DC-specific deletion of Klotho did not affect kidney ROMK expression (Supplementary Figure S21A) or serum and urinary K^+^ levels (Supplementary Figure S21B). This may be due to the plethora of mechanisms that maintain potassium homeostasis.

A major strength of the present study lies in revealing the critical role of the DC in sKlotho secretion into urine and regulating Ca^2+^ reabsorption, demonstrated using 3 new mouse models. Nonetheless, we acknowledge the limitations of our study. Although all available evidence from the present study supports the notion that the PT is the source of circulating sKlotho, additional experiments are needed to confirm this unequivocally. Secondly, the physiological or clinical relevance of sKlotho in normal urine is currently lacking. In preliminary studies, we observed that urinary sKlotho levels exceed serum levels (Supplementary Figure S22), suggesting that abundant sKlotho levels in urine might impact the kidneys or urinary tract.

In conclusion, our study identified critical roles of Klotho in DC in regulating urinary sKlotho levels and Ca^2+^ reabsorption, distinct from PT where Klotho primarily regulates Pi homeostasis, and likely contribute to circulatory sKlotho levels. Therefore, our findings suggest that Klotho in the kidney have segment-specific divergent roles in the PT and DC.

## Supplementary Material

1

2

Supplementary material is available online at www.kidney-international.org.

## Data Availability

All data are available from the authors on reasonable request. The raw bulk RNA-seq data used in this publication have been deposited in NCBI’s Gene Expression Omnibus and are accessible through GEO Series accession number GSE247008 (https://www.ncbi.nlm.nih.gov/geo/query/acc.cgi?acc=GSE247008).

## Translational Statement

Klotho regulates mineral metabolism, is cardio- and renoprotective, and extends lifespan in mice. Some of these effects are attributed to soluble Klotho (sKlotho), present in blood and urine and derived from cleavage of membrane-bound Klotho. Klotho is highly expressed in the kidney, where it is found in both the proximal and distal tubules. In the present study, we used single-cell RNA sequencing and 4 renal tubule–specific Klotho knockout mouse models to demonstrate that the renal distal convolution (DC) is the primary source of urinary sKlotho. DC-derived Klotho is critical for renal calcium transport and bone health but has no impact on tubular phosphate (Pi) handling, whereas Klotho in proximal tubule maintains Pi homeostasis and likely regulates circulating sKlotho levels. This study comprehensively dissects the biology of Klotho derived from different nephron segments.
