# Supplementary material for: Klotho in the kidney distal convolution regulates urinary Klotho excretion and kidney calcium reabsorption, but not phosphate homeostasis
Source: Kidney Int. Author manuscript; Available in PMC 2026 Jun 25. (PMC13294757; doi:10.1016/j.kint.2026.01.030)
Supplement: 1 [file NIHMS2178793-supplement-1.pdf]

Klotho in the kidney distal convolution regulates urinary Klotho excretion and kidney calcium reabsorption, but not phosphate homeostasis.

Laurent Bourqui[1,2], Adisa Trnjanin[1], Klaudia Kopper[1,2], Dominique Loffing-Cueni[1], Zsuzsa Radvanyi[1,2], Artyom Karpovich[1], Tara Rahimi[1], Rui Santos[1], Agnieszka Wengi[1], Johanne Pastor[3], Orson W. Moe[3, 4], Johannes Loffing[1,2,6#], Ganesh Pathare[1,2,5,6#]

[1]Institute of Anatomy, University of Zürich, Zürich, Switzerland; [2]Swiss National Centre of Competence in Research "Kidney Control of Homeostasis", Switzerland; [3]Charles and Jane Pak Center for Mineral Metabolism and Clinical Research, The University of Texas Southwestern Medical Center, Dallas, TX, United States; [4]Departments of Internal Medicine and Physiology, The University of Texas Southwestern Medical Center, Dallas, TX, United States; [5]Bone-Kidney Axis and Regeneration Laboratory, Dept. of Infectious Diseases and Public Health, Jockey Club College of Veterinary Medicine and Life Sciences, City University of Hong Kong, Hong Kong SAR

[6]Contributed equally

[#]Correspondence:
Prof. Johannes Loffing, MD
Institute of Anatomy
University of Zürich
Winterthurerstrasse 190, 8057, CH
Email: johannes.loffing@anatomy.uzh.ch

Prof. Ganesh Pathare, BVSc, PhD
Bone-Kidney Axis and Regeneration Laboratory
Dept. of Infectious Diseases and Public Health
Jockey Club College of Veterinary Medicine and Life Sciences
City University of Hong Kong
To Yuen Street 31, 999077, Hong Kong SAR
Email: gpathare@cityu.edu.hk

Competing Interests

The authors declare no potential conflicts of interest.

Running title

Segment-specific roles of Klotho in the kidney

This PDF file includes:

Supplementary figures with figure legends

Supplementary tables

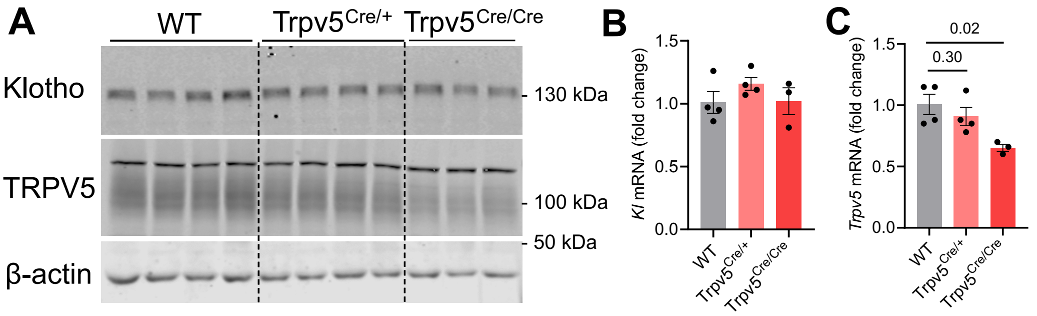

D

	$Trpv5^{wt}$ (n=4; male)	$Trpv5^{Cre/+}$ (n=4; male)	$Trpv5^{Cre/Cre}$ (n=3; male)	p-value
Body weight	26.13 (± 1.132)	26.95 (± 1.193)	24.57 (± 0.681)	0.8027
pH	7.3 (±0.13)	7.4 (±0.01)	7.4 (±0.03)	
Hct (%)	42.50 (± 2.38)	41.25 (± 0.96)	40 (± 3.61)	0.5344
cNa^{+} (mmol/l)	147.0 (± 2.16)	147.3 (± 0.957)	146.7 (± 0.577)	0.3138
cK^{+} (mmol/l)	3.735 (± 0.187)	3.783 (± 0.371)	4.033 (± 0.149)	0.3977
cCa^{2+} (mmol/l)	1.263 (± 0.193)	1.328 (± 0.060)	1.17 (± 0.105)	0.3563
cCl^{-} (mmol/l)	106 (± 1.414)	107 (± 1.633)	107.7 (± 0.577)	0.3112

Fig. S1. Effect of genetic modification in *Trpv5* gene on TRPV5 expression, bodyweight and blood parameters. A) Immunoblot of Klotho, TRPV5 and β-actin in total kidney lysate obtained from 8-12 weeks old male wild-type, *Trpv5*$^{Cre/+}$ and *Trpv5*$^{Cre/Cre}$ mice. (n=3-4, each group). **B)** Renal *Kl;* **C)** *Trpv5* mRNA levels normalized to *Tbp* in 8-12 weeks old male wild-type, *Trpv5*$^{Cre/+}$ and *Trpv5*$^{Cre/Cre}$ mice; **D)** Body weight and blood parameters. (n=3-4, each group).

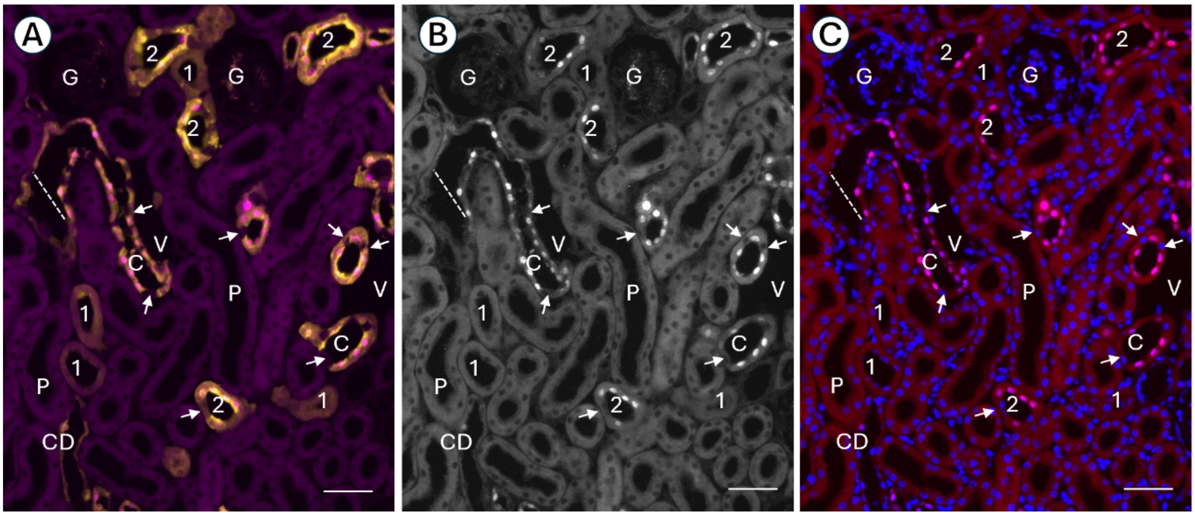

Fig. S2: Localization of nuclear Cre staining in the kidneys of tamoxifen-treated *Trpv5*$^{Cre/+}$ mice is exclusively observed in principal cells of the DCT2 and CNT. A) Double staining for Cre (magenta) and CaBP28K (yellow). **B)** Staining for Cre only. **C)** Double staining for Cre (red) and DAPI (blue). Colocalization of Cre and DAPI results in a magenta appearance of the nucleus. Arrows indicate cre negative intercalated cells. The transition from CNT to CD is indicated by a dashed line. Scale bar: ~50 µm. G = glomerulus; P = Proximal tubule; 1 = DCT1; 2 = DCT2; C = CNT; CD = collecting duct; V = vein.

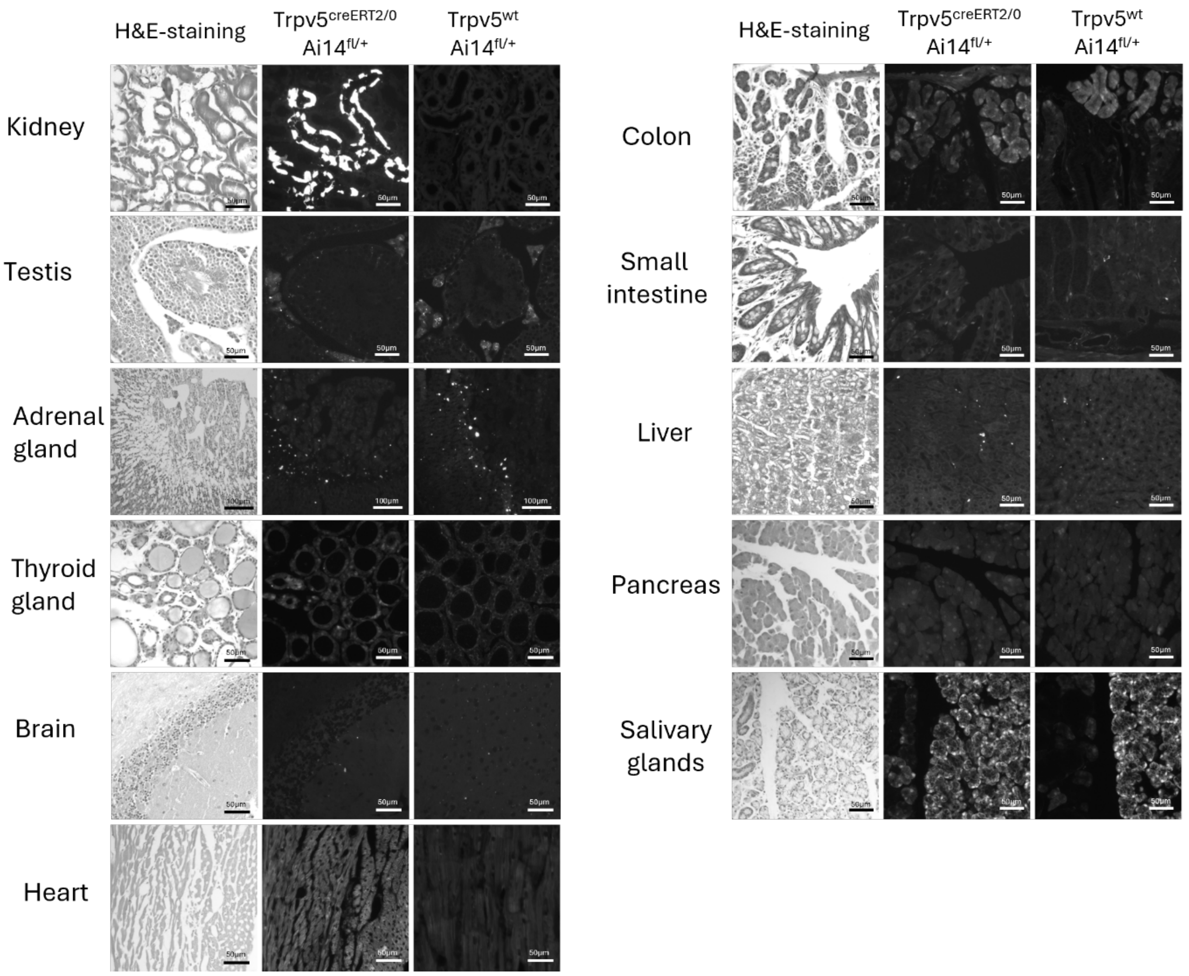

Fig. S3 Analysis of tdTomato expression in various organs of Ai14-Reporter mice heterozygous for Trpv5^{Cre} (Trpv5$^{creERT2/0}$Ai14$^{fl/+}$) or wildtype for Trpv5^{Cre} (Trpv5^{wt}Ai14$^{fl/+}$). Tamoxifen-treated mice heterozygous for Trpv5^{Cre} express tdTomato protein exclusively in the kidney. No tdTomato signal was detected in other analyzed organs of these mice. In Trpv5^{wt} mice, no tdTomato expression was detected in the kidney or any other analyzed organs.

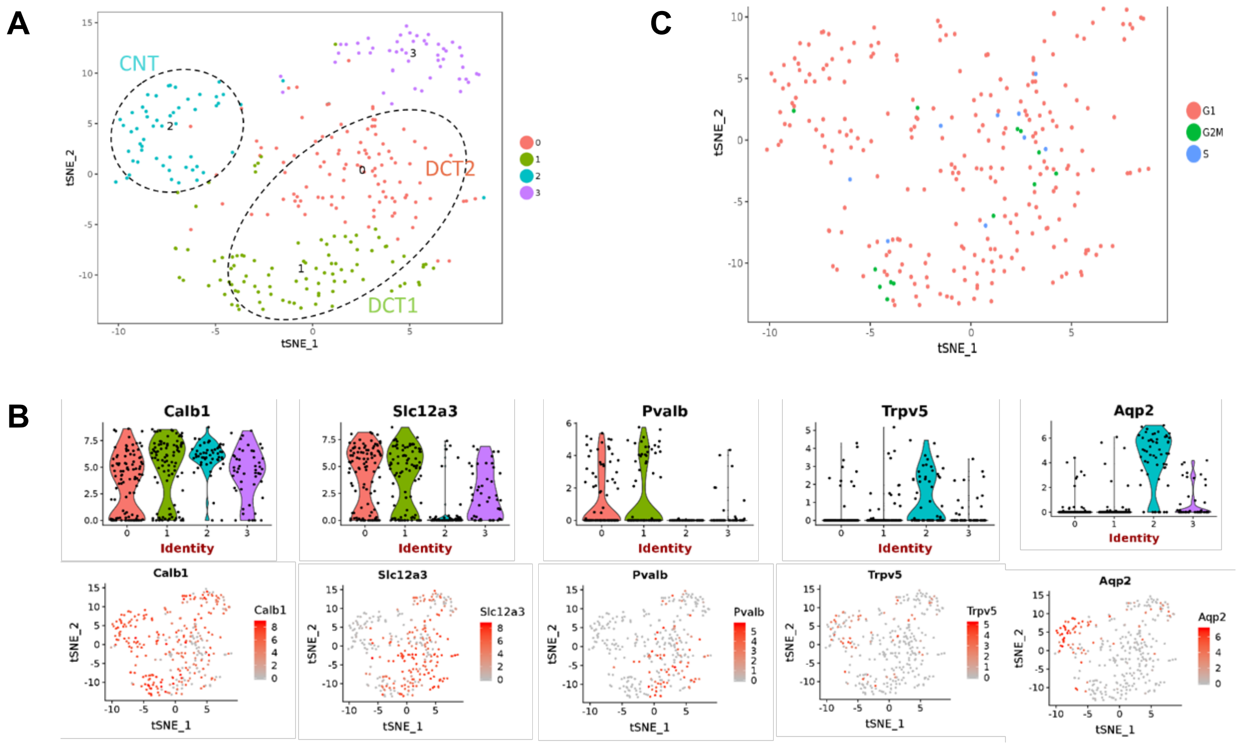

Fig. S4: *scRNA-seq of DC. Distance metrics driven, unsupervised clustering based on principal component analysis reveals 4 distinct cell clusters. A)* *Unsupervised clustering reveals 4 clusters identified as 3 cell types shown in tSNE map (tSNE_1 = principal component 1; tSNE_2 = principal component 2; color-coded cell cluster identity).* ***B)*** *Clusters were identified based on expression of known marker genes. Clusters were identified as DCT2 cells in Cluster 0 (orange; 106 cells); DCT1 cells in Cluster 1 (green; 93 cells); PC-CNT cells in Cluster 2 (blue; 55 cells). Cluster 3 cells (purple; 49 cells) remain unidentified cell types (Upper panel: x-axis = cluster identity; y-axis = expression levels; lower panel: x-axis = principal component 1; y-axis = principal component 2)* ***C)*** *Cell cycle analysis reveals a small proliferating population in DCT2 and DCT1 cells. The cell cycle analysis was performed bioinformatically using Seurat based on the expression values of proliferation markers (G1 = G1-phase; G2M = G2 and M-phase; S = S-phase of the cell cycle).*

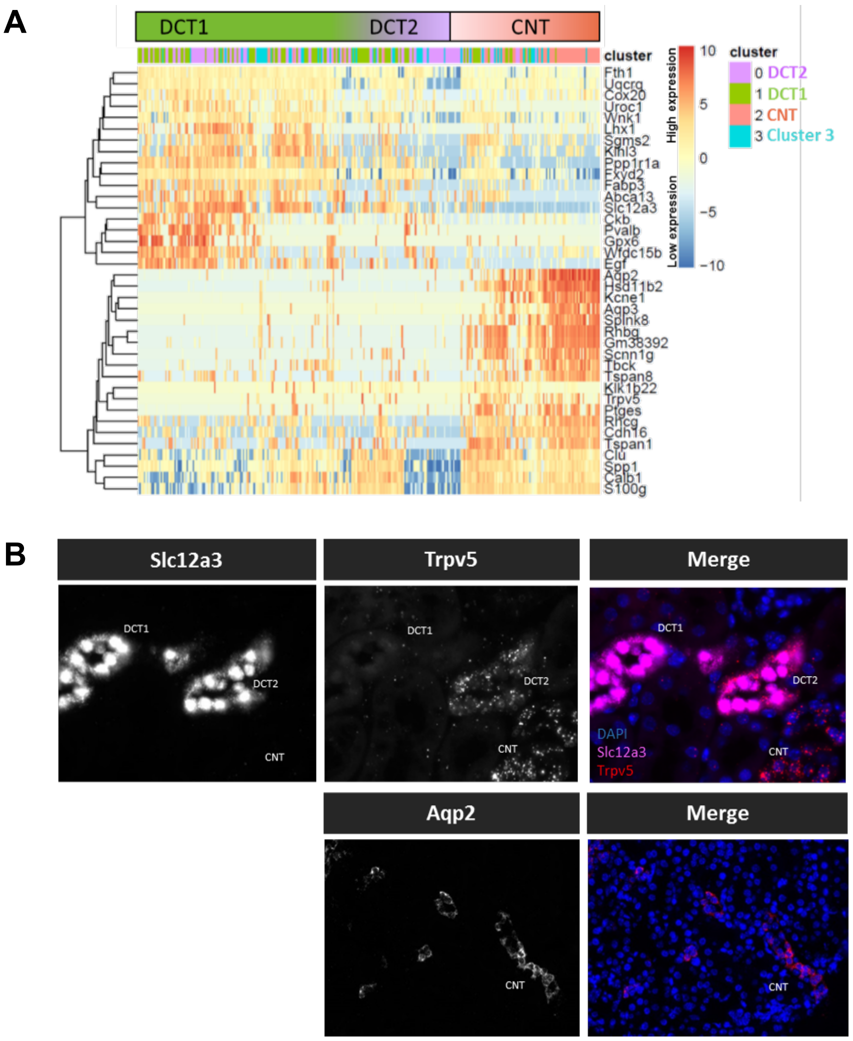

Fig. S5: *scRNA-seq of DC: Gradient analysis and ISH confirmation based on single cell transcriptome of DCT and CNT cells. A)* *Heat map of relative gene expression (red, high; blue, low) in 4 cell clusters after transcriptome analysis by Seurat.* ***B)*** *Fluorescence microscopy of kidney tissue ISH: Expression of Slc12a3, Trpv5 and Aqp2 mRNA. Areas of CNT, DCT1 and DCT2 are indicated. ISH with RNAscope Assay, upper right: merge of Slc12a3 and Trpv5, lower right: merge of Aqp2 and DAPI. 40x magnification.*

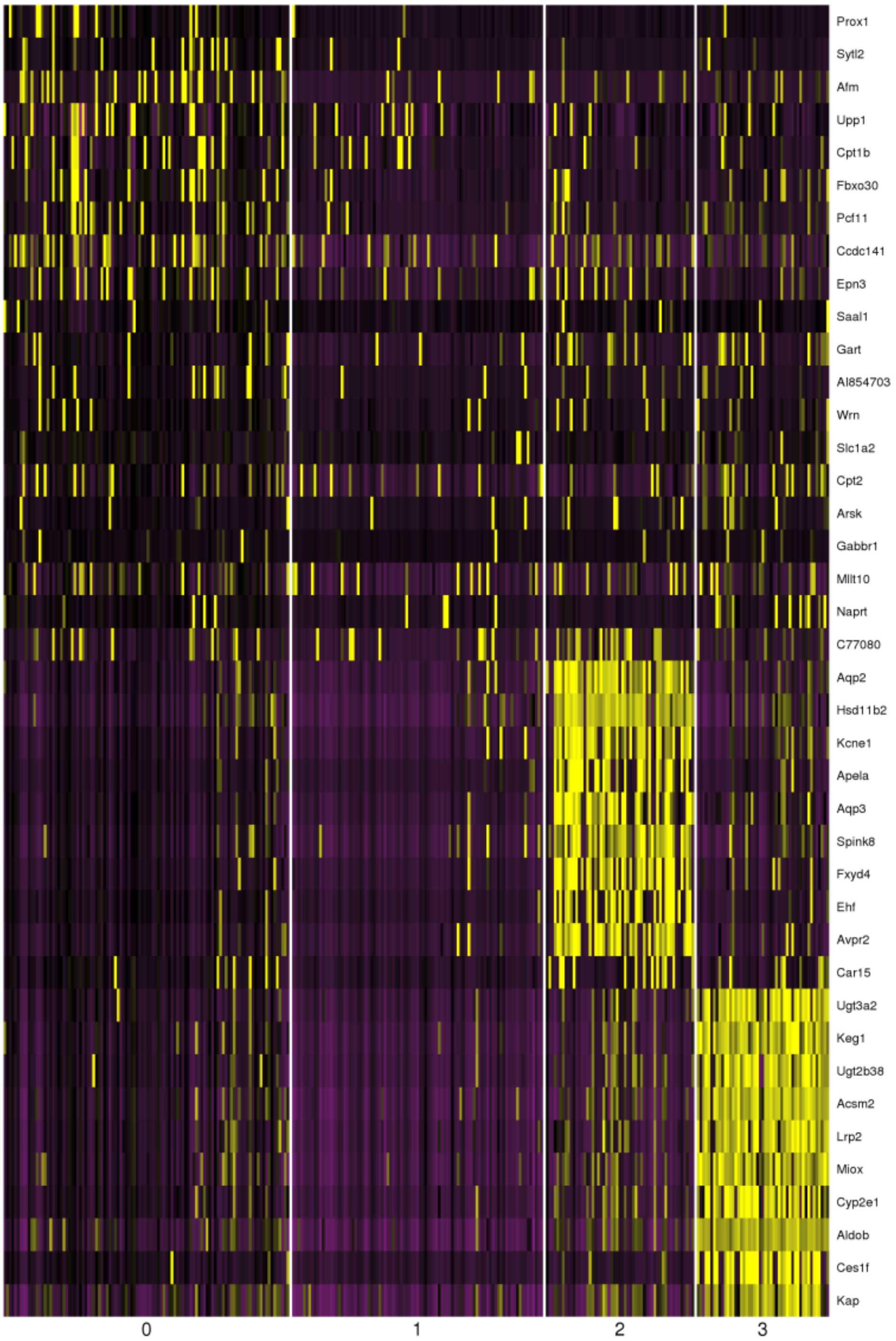

Fig. S6: scRNA-seq of DC: Heat map shows top 10 positive markers per cluster. Yellow color represents strong gene expression. Labelling X axis shows Clusters 0-3, Y axis shows 10 highly expressed marker genes for each cluster.

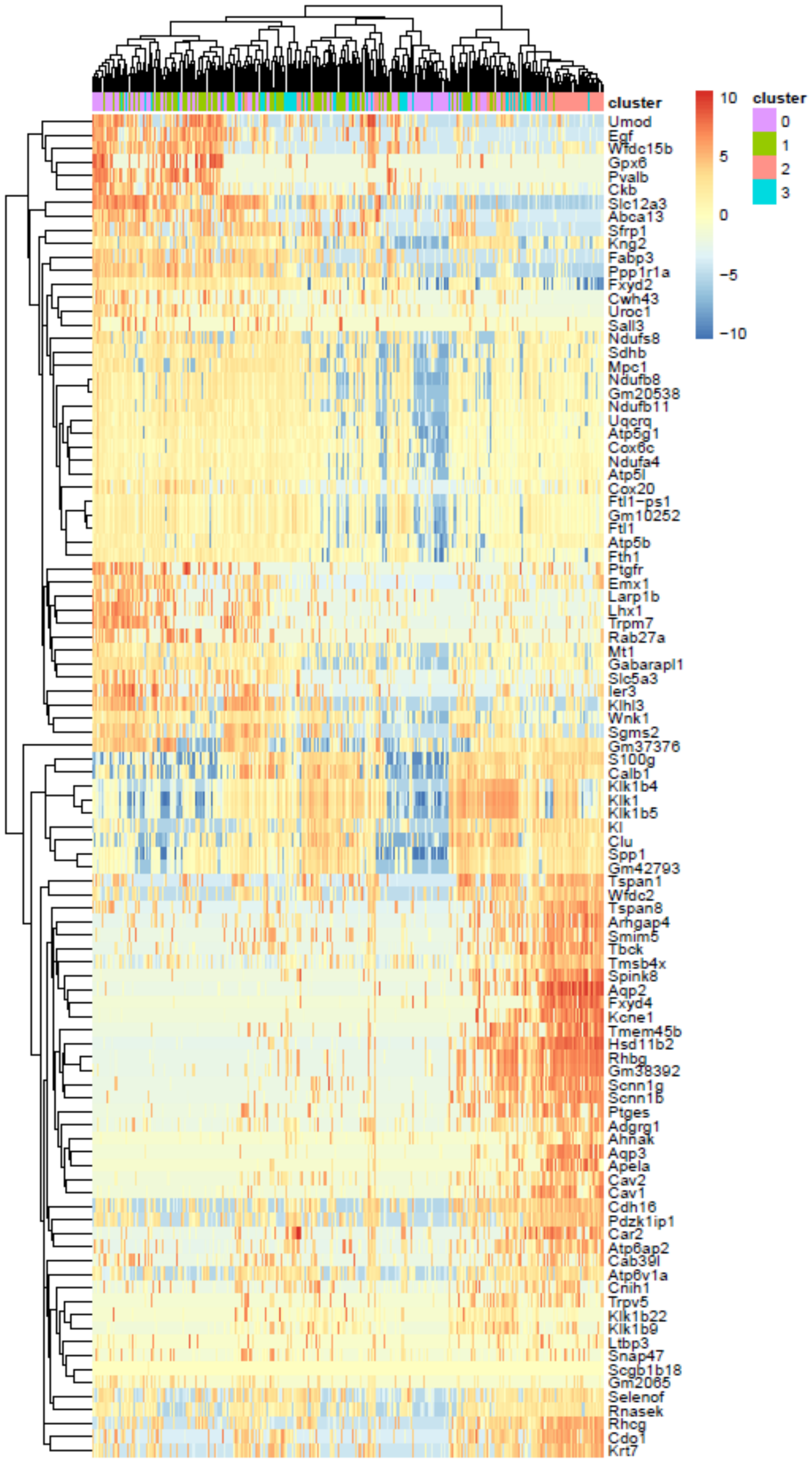

Fig. S7: ***scRNA-seq of DC: Heatmap representing gradient map.*** *Labeling: x-axis shows clustering of the cells (Cluster identity is color coded); y-axis left side shows gene clustering, y-axis right side shows gene names.*

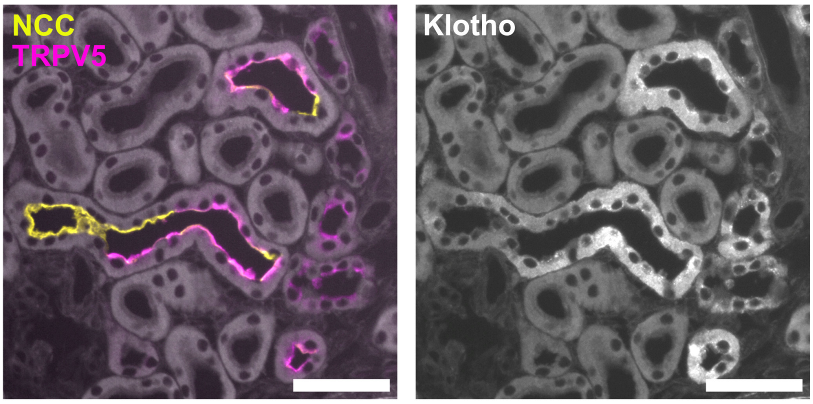

Fig. S8. Klotho is primarily enriched in DCT2/CNT. Immunofluorescence on consecutive kidney sections showing TRPV5 in magenta and NCC in yellow (left panel), while Klotho is shown in the right panel. Note higher Klotho expression in TRPV5 positive DCT2/CNT cells rather than NCC (pNCC-58) positive DCT cells. Scale bar: ~50 μm.

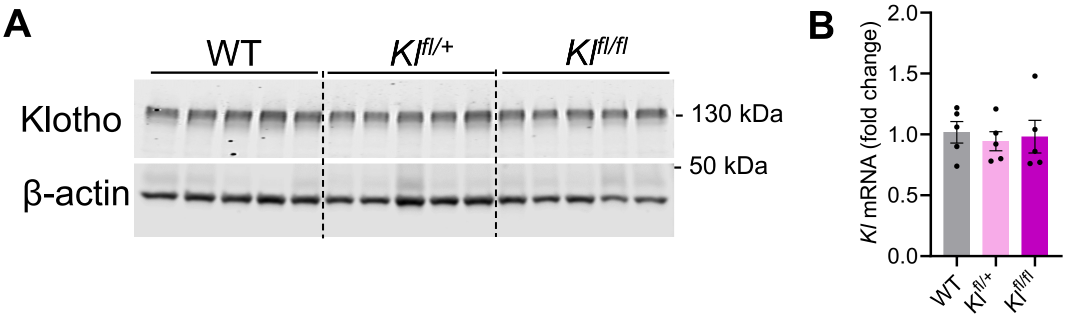

Fig. S9. Klotho flox mice display normal Klotho protein and mRNA expression. A) Original immunoblot of Klotho and β-actin in total kidney lysate obtained from 8-12 weeks old male wild-type, *Kl*$^{fl/+}$ and *Kl*$^{fl/fl}$ mice. (n=5, each group). **B)** Renal *Kl* mRNA levels normalized to *Tbp* in 8-12 weeks old male wild-type, *Kl*$^{fl/+}$ and *Kl*$^{fl/fl}$ mice. (n=5, each group).

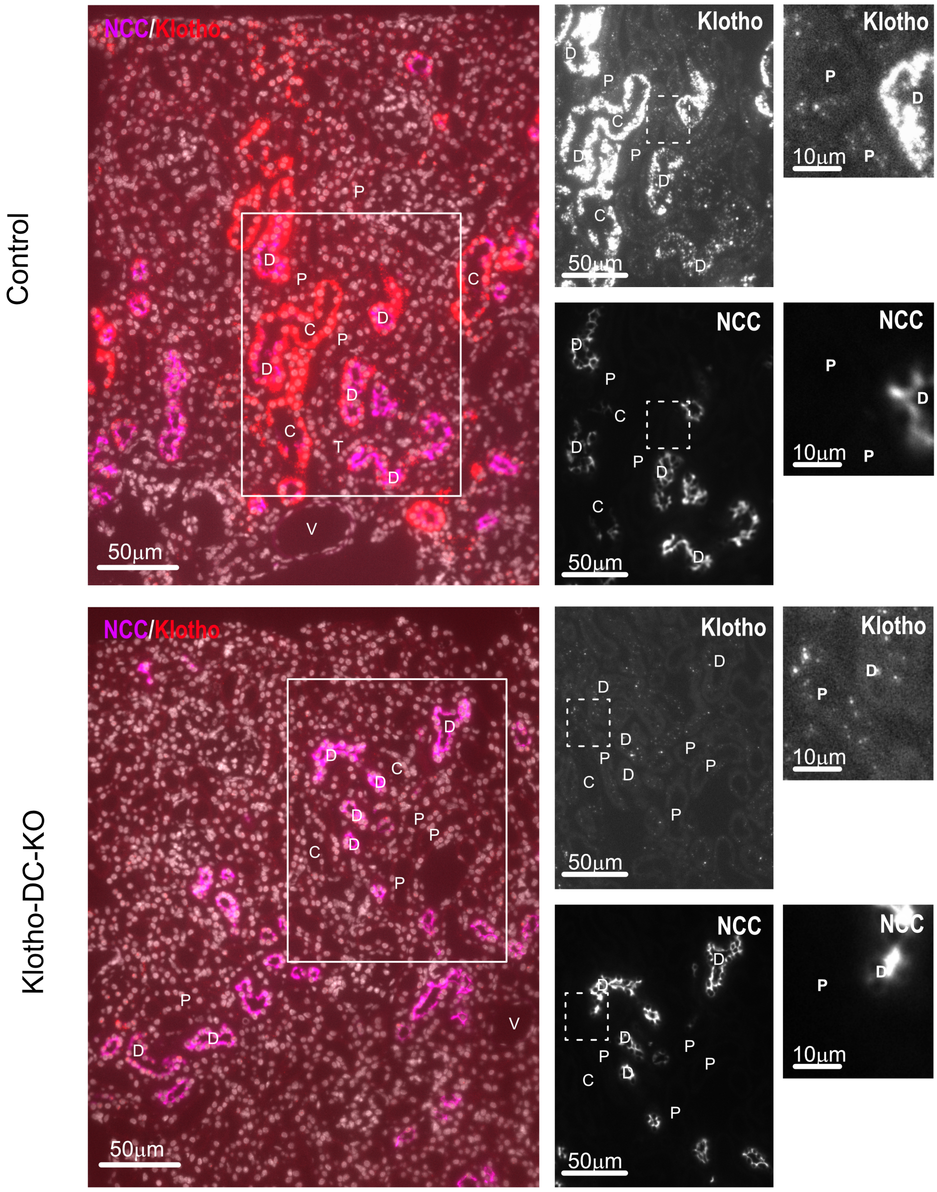

Fig. S10. Confirmation of DC-specific Klotho deletion by RNAscope. Representative kidney sections from control (upper panels) and Kl-KO^{DC} mice (lower panels) are shown. Klotho mRNA is detected using RNAscope probes (red), while NCC is detected using immunofluorescence (magenta). Labels indicate nephron segments: P- proximal tubule; D- distal convolution; C- connecting tubule; V- vessel. Nuclei are counterstained with DAPI (gray/white). Boxed regions indicate areas shown at higher magnification on the right. In control kidneys, robust Klotho mRNA expression is observed in NCC-positive DC, whereas Klotho signal is markedly reduced/absent in DC of Kl-KO^{DC} mice. Klotho mRNA expression in proximal tubule is weak and similar in both control and Kl-KO^{DC} mice. Of note, our targeting strategy deletes exon 2 of the *Klotho* gene and introduces a frameshift resulting in an early stop codon, which explains the absence of Klotho protein detection by immunofluorescence (Figure 2B). The RNAscope probe used in our study binds to the regions of *Klotho* mRNA, which extend beyond exon 2. Thus, the weak residual RNAscope signal observed in the DC of DC-specific Klotho knockout mice likely represents exon 2 deleted but not fully degraded RNA. Scale bars: 50 µm (overview images) and 10 µm (high magnifications).

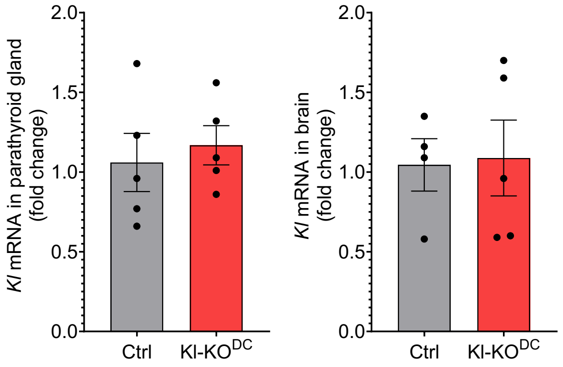

Fig. S11. Unchanged extra-renal *Kl* levels in Kl-KO^{DC} mice. *Kl* mRNA levels normalized to *Tbp* in parathyroid glands (left panel) and brain (right panel) in 8-weeks old male wild-type and Kl-KO^{DC} mice. (n=5, each group).

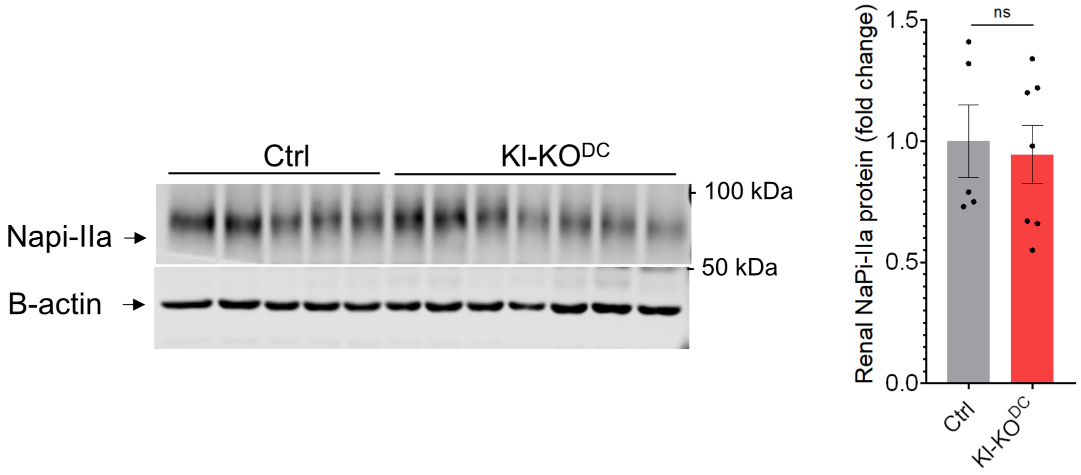

Fig. S12. Left panel: Original immunoblot of NaPi-IIa in total kidney lysates of Kl-KO^{DC} and corresponding matching control mice. **Right panel:** Densitometric analysis of NaPi-IIa normalized to β-actin (8-10 weeks old male mice; n=5-7, each group).

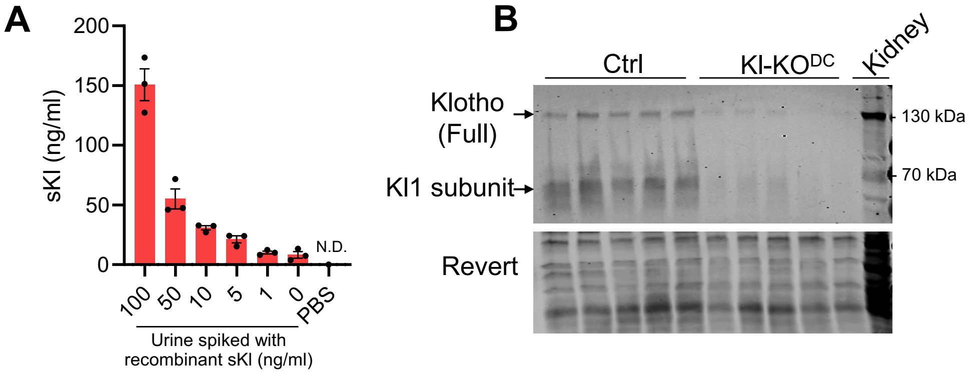

Fig. S13. Validation of urinary sKlotho measurements in mice. A) The commercial ELISA was validated by measuring sKlotho in urine samples from 8-week-old, wild-type male mice (n=3 per group), which were spiked with recombinant sKlotho. **B)** Original immunoblot displaying both full-length sKlotho and the sKlotho subunit (Kl1) in the 24-h urine samples from 8-week-old, wild-type male mice (n=5 per each group). Total kidney lysate served as a positive control. While revert stain confirming equal loading.

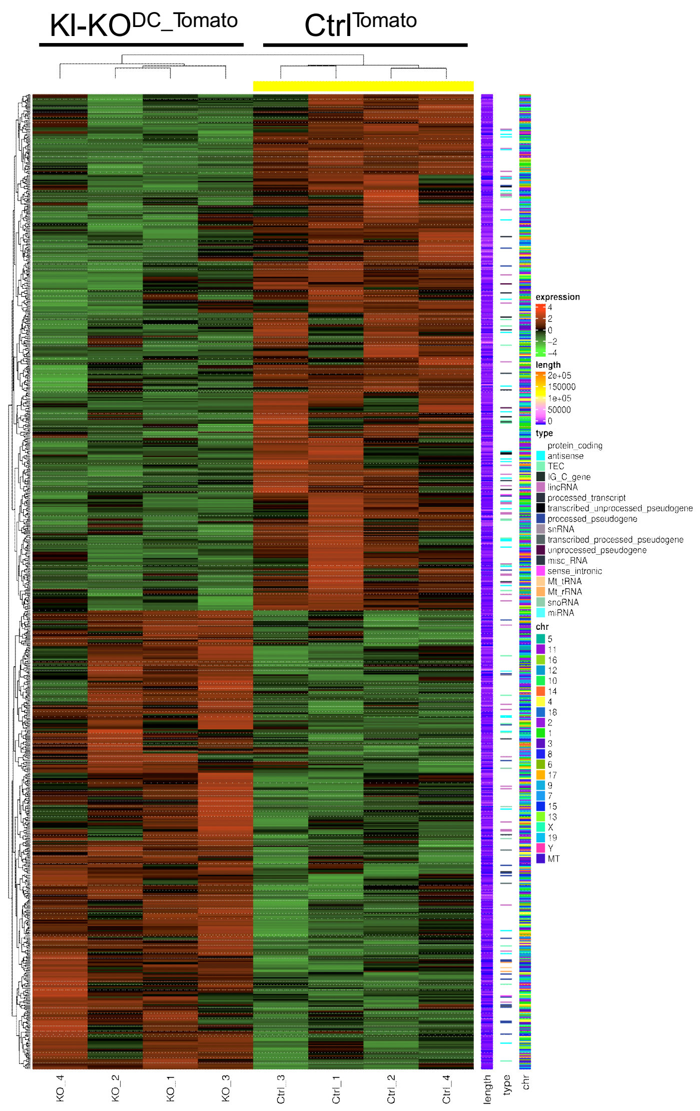

Fig. S14. Heatmap of gene expression levels in DC cells isolated from control and Kl-KO^{DC} mice.

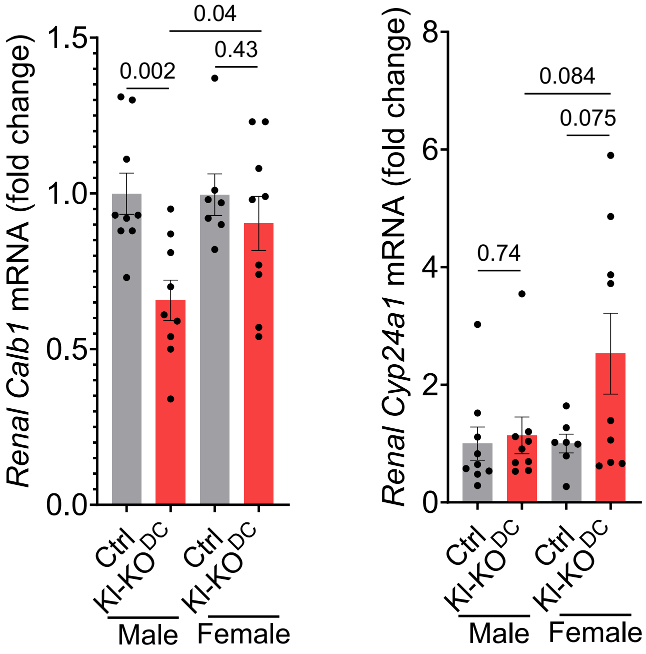

Fig. S15. Renal *Cyp24a1 and Calb1* mRNA levels normalized to *Tbp* in control and KI-KO^{DC} mice. (n=7-9 per group).

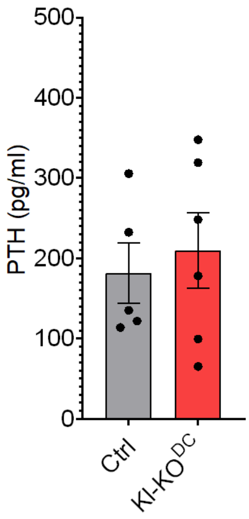

Fig. S16. Serum PTH levels in KI-KO^{DC} and corresponding matching control mice. (8-10 weeks old male; n=5-6, each group).

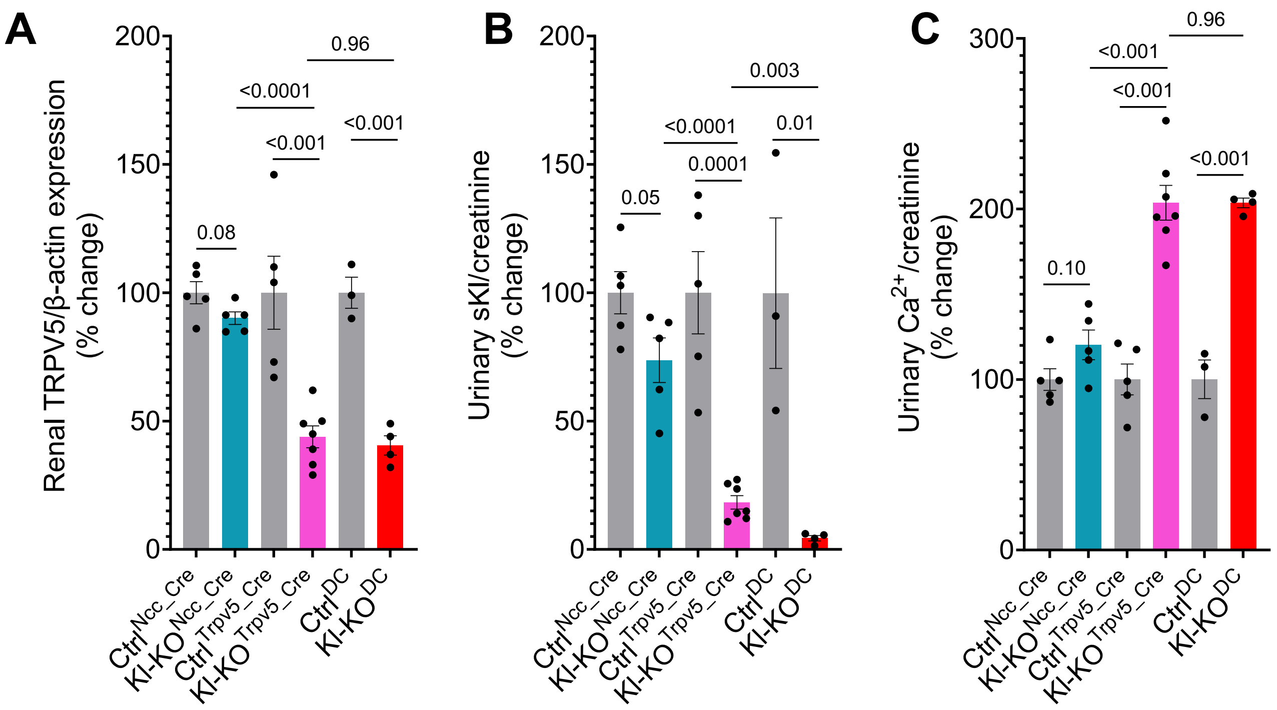

Fig. S17. Comparison of urinary sKlotho levels and Ca^{2+} homeostasis in Kl-KO^{Ncc_Cre}, Kl-KO^{Trpv5_Cre}, Kl-KO^{DC} A) Densitometric analysis of TRPV5 normalized to β-actin in Kl-KO^{Ncc_Cre}, Kl-KO^{Trpv5_Cre}, Kl-KO^{DC}, and corresponding control mice (n=3-7 mice per group). **B)** sKlotho levels in 24-hour urine, normalized to creatinine in Kl-KO^{Ncc_Cre}, Kl-KO^{Trpv5_Cre}, Kl-KO^{DC}, and corresponding control mice (n=3-7 mice per group). **C)** Urinary Ca^{2+} excretion measured in 24-hour urine, normalized to creatinine in Kl-KO^{Ncc_Cre}, Kl-KO^{Trpv5_Cre}, Kl-KO^{DC}, and corresponding control mice (n=3-6 mice per group).

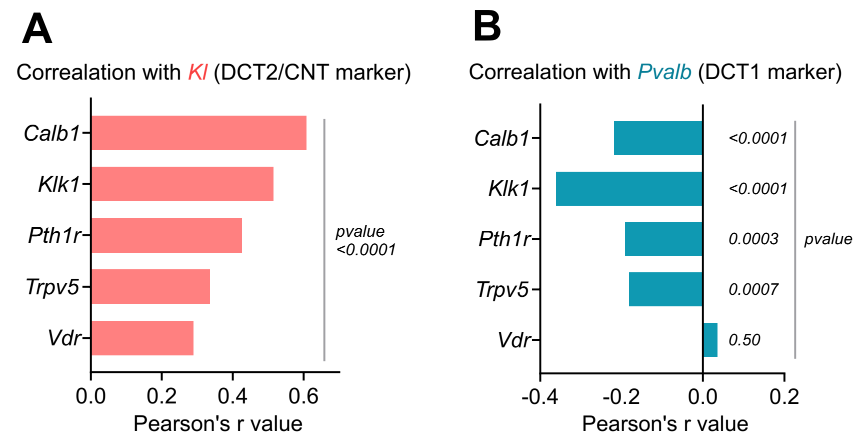

Fig. S18. Klotho targets involved in Ca^{2+} reabsorption are strongly enriched in DCT2/CNT than DCT1. A) Correlation analysis between *Kl* and *Calb1, Klk1, Pth1r, Trpv5* and *Vdr.* The X axis depicts r value (p<0.0001). **B)** Correlation analysis between *Pvalb* and *Calb1, Klk1, Pth1r, Trpv5* and *Vdr.* The X axis depicts r value (p values are denoted for individual analysis).

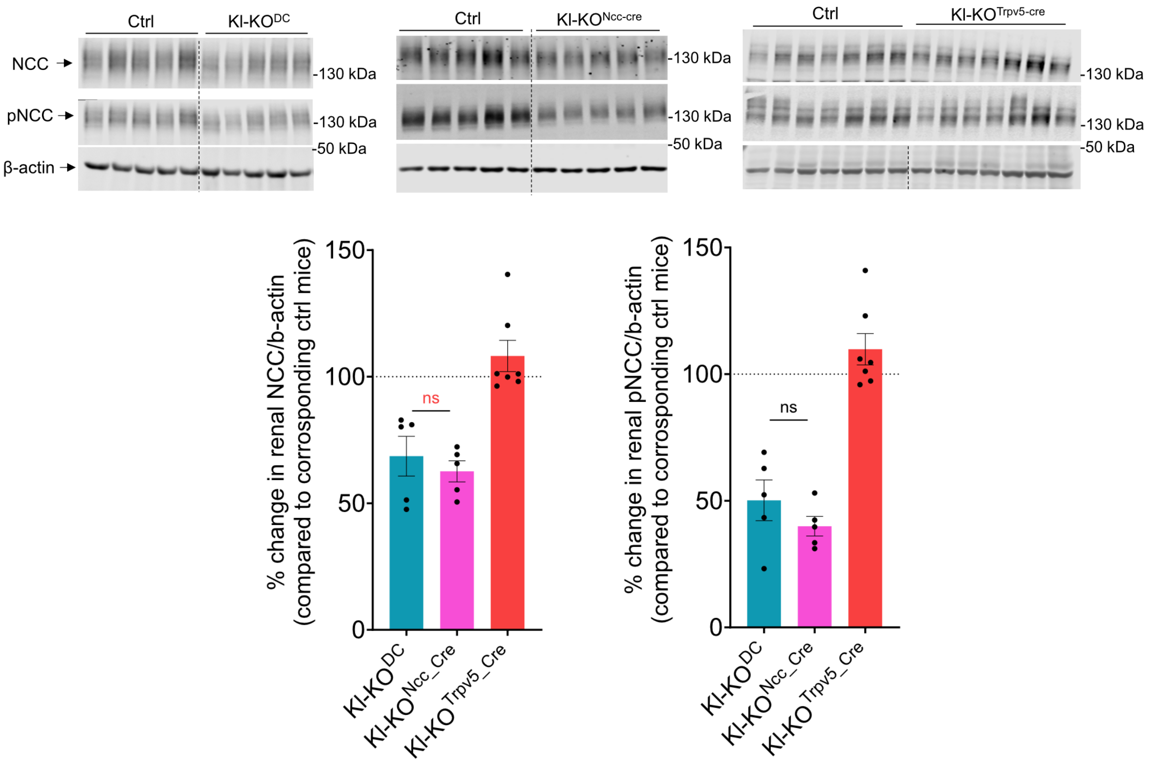

Fig. S19. NCC expression levels in total kidney lysates of Kl-KO-DC, Ncc-Cre, Trpv5-Cre and corresponding control mice. Upper panels: immunoblots, lower panels: corresponding densitometry. (n=5-7 male mice per group)

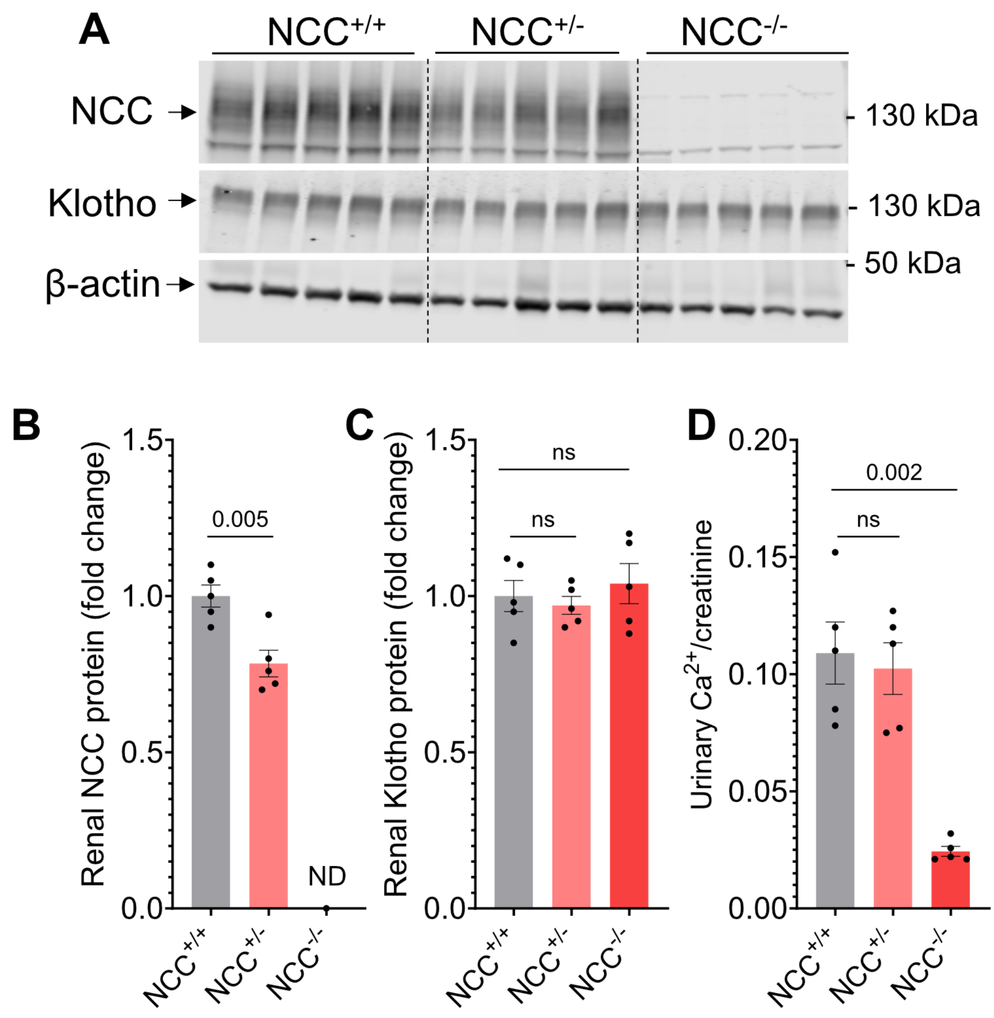

Fig. S20. Global NCC knockout mice exhibit hypocalciuria, while Klotho expression in total kidney lysates remains unchanged. **A)** Immunoblots for NCC and Klotho proteins in wild-type, heterozygous, and homozygous NCC knockout mice (10–12-week-old, male; n = 5, per group). Corresponding densitometric analyses for NCC **B)** and Klotho **C)**. **D)** Urinary calcium excretion normalized to creatinine.

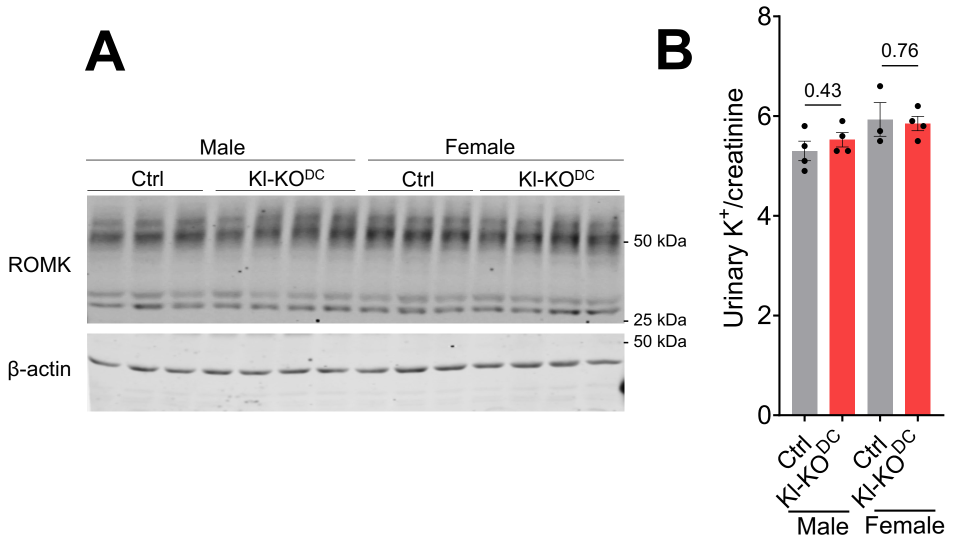

Fig. S21. Unchanged potassium homeostasis in Kl-KO^{DC} mice. A) Original immunoblot of ROMK and β-actin in total kidney lysate obtained from 8-12 weeks old control and Kl-KO^{DC} mice. (n=3-4, each group). **B)** K^+ excretion in 24-h urine, normalized to creatinine (n=3-4, each group).

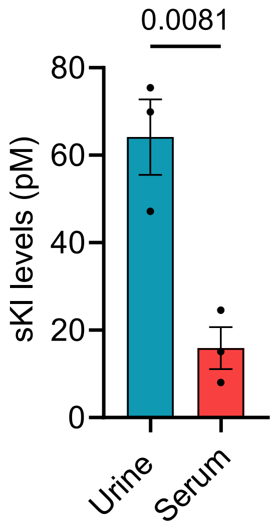

Fig. S22. Urinary levels of sKlotho exceed serum levels. The urine and serum samples from the same three mice were analyzed for sKlotho levels using the IP-IB method.

Nr.	Mouse model	Nomenclature	Description	Purpose	Phenotype	Provider, reference
			**Parental lines**			
1	**Ncc$^{Cre/+}$**	B6-Slc12a3<tm(cre/ERT2)Jlo>	Cre-recombinase in the 3'-UTR of the *Slc12a3* gene	To target DCT cells	None	J. Loffing, (doi: 10.1007/s00424-020-02491-1)
2	**Trpv5$^{Cre/+}$**	B6-Trpv5<tm(cre/ERT2)Jlo>	Cre-recombinase in the 3'-UTR of the *Trpv5* gene	To target DCT2/CNT cells	None	J. Loffing, (this study)
3	**Pax8-rTA/$^{LC1Cre/+}$**	B6.Cg-Tg(Pax8-rtTA2S*M2)1Koes/J	Pax8-rtTA-mediated, doxycycline controlled Cre-recombinase expression	To target all tubule epithelial cells	None	R. Koesters, (doi:10.1038/nm.1865)
4	**Rosa26^{tdT} /Ai14**	B6.Cg-*Gt(ROSA)26Sortm14(CAG-tdTomato)Hze*/J	Tomato reporter mice	To express red fluorescent tdTomato protein conditionally	None	H. Zeng, (doi:10.1038/nn.2467)
5	**Klotho-Flox** (Kl$^{fl/fl}$)	B6-Klotho<tm1Jlo>	loxP sequences were inserted flanking exon 2 of *Kl gene*	To knock out Klotho conditionally	None	J. Loffing, (this study)
			**Combinatory lines**			
6	**Ncc$^{Cre/+}$Rosa26^{tdTomato}**		Rosa26^{tdT} mice crossed with Ncc$^{Cre/+}$ mice	To isolate fluorescent DCT cells	Red fluorescent DCT cells	
7	**Trpv5$^{Cre/+}$Rosa26^{tdTomato}**		Rosa26^{tdT} mice crossed with Ncc$^{Cre/+}$ mice	To isolate fluorescent DCT2/CNT cells	Red fluorescent DCT2/CNT cells	
8	**Kl-KO$^{Ncc\text{-}Cre}$** (Kl$^{fl/fl}$Ncc$^{Cre/+}$)		Kl$^{fl/fl}$ mice crossed with Ncc$^{Cre/+}$ mice	To knock out Klotho in DCT cells	Subtle hypercalciuria, slight reduction in urinary sKlotho excretion	
9	**Kl-KO$^{Trpv5\text{-}Cre}$** (Kl$^{fl/fl}$Trpv5$^{Cre/+}$)		Kl$^{fl/fl}$ mice crossed with Trpv5$^{Cre/+}$ mice	To knock out Klotho in DCT2/CNT cells	Hypercalciuria, significant reduction in urinary sKlotho excretion	
10	**Kl-KO^{DC}** (Kl$^{fl/fl}$Ncc$^{Cre/+}$Trpv5$^{Cre/+}$)		Kl$^{fl/fl}$ mice crossed with Ncc$^{Cre/+}$ and Trpv5$^{Cre/*}$ mice	To knock out Klotho in all DC cells	Hypercalciuria, almost abolished urinary sKlotho excretion, reduced bone density	
11	**Kl-KO^{DC_Tomato}** (Kl$^{fl/fl}$Ncc$^{Cre/+}$Trpv5$^{Cre/+}$ Tomato$^{Tg/+}$)		Kl$^{fl/fl}$ mice crossed with Ncc$^{Cre/+}$, Trpv5$^{Cre/+}$ and Rosa26^{tdT} mice	To isolate fluorescent DC cells lacking Klotho	Not determined	
12	**Kl-KO^{Kidney}** (Kl$^{fl/fl}$Pax8-rTA/LC1)		Kl$^{fl/fl}$ mice crossed with Pax8-rTA/LC1$^{cre/+}$ mice	To knock out Klotho in all tubule epithelial cells including PT and DC	Severe hyperphosphatemia, hypercalcemia, and loss in body weight	

Table S1. Summary of the mouse models used in the present study

#*Mouse lines 3 and 12 (*Pax8-rTA/$^{LC1Cre/+}$, *Kl-KO^{Kidney}) are doxycycline-inducible models, whereas all other cre-mouse lines (1,2, and 6-11) are tamoxifen-inducible models.*

Primer		**Sequence 5'-3'**
Klotho-fl	fwd.	TAT GTA ACA CTG CTC TCA TTT GGG
	rev.	TGA AAG AGG GAG CTA CTG GTG GTA G
Ncc^{cre}	fwd. 1	CTG ATG CTC TTC CTC CTA CAG TAC
	fwd. 2	CAG CTC CTC CTC ATC CTC TCC CAC A
	rev.	GGA AAC CCC ATC TTC TGC ATA GG
$Trpv5^{cre}$	fwd. 1	CTG GGA CAT TTG AAT CTT GGA C
	fwd. 2	GTA CAG CAT GAA GTG CAA GAA C
	rev.	AGG ATA TGA GCT TTA GAG GAA CTG

Table S2. List of primers used for genotyping mice in the present study

**Antibody**	**Host**	**Dilution WB**	**Dilution IF**	**Reference**
Anti-Klotho (Kl1, clone: KM2076)	Rat	1:1'000	-	TransGenic; Cat. #KO603
Anti-Klotho	Goat	-	1:200	R&D Systems; Cat. #AF1819
Anti-pT58NCC	Rat	-	1:5’000	(Banki et al., 2021) doi: 10.1016/j.kint.2021.06.030
Anti-ROMK	Rabbit	1:1’000	-	(Penton et al., 2020) doi:10.1016/j.kint.2019.12.016
Anti-total NCC	Rabbit	1:5’000	1:5’000	(Sorensen et al., 2013) doi:10.1038/ki.2013.14
Anti-TRPV5	Rabbit	1:1’000	1:5’000	(Moor et al., 2018) doi:10.3389/fphys.2018.00874
Anti-β-actin	Mouse	1:10'000	-	Santa Cruz; Cat. #sc-47778X
AF488-conjugated-anti-goat	Donkey	-	1:1'000	Thermo Fisher; Cat. # A11055
AF555-conjugated-anti-rat	Donkey	-	1:1'000	Thermo Fisher; Cat. # A48270
AF647-conjugated-anti-rabbit	Donkey	-	1:1'000	Jackson Immu.; Cat. # 711-605-152
IRDye680-conjugated anti-mouse	Goat	1:10'000	-	LI-COR; Cat. # 926-68070
IRDye800-conjugated anti-Goat	Donkey	1:10'000	-	LI-COR; Cat. # 926-32214
IRDye800-conjugated anti-rabbit	Goat	1:10'000	-	LI-COR; Cat. # 926-32211
IRDye800-conjugated anti-Rat	Goat	1:10'000	-	LI-COR; Cat. # 926-32219

Table S3. List of antibodies used in the present study

Primer		Sequence 5'-3'
Calb1	fwd.	ATT TCG ACG CTG ACG GAA GT
	rev.	CCA ATC CAG CCT TCT TTC GC
Cyp24a1	fwd.	TGG GCT CTA GCG AAG ACA AT
	rev.	GGT ACC AGG ATG CCA AGA TG
Cyp27b1	fwd.	CCG CGG GCT ATG CTG GAA C
	rev.	CTC TGG GCA AAG GCA AAC ATC TGA
NaPi-IIa	fwd.	TCA GGA AGA GGA GCA AAA GC
	rev.	AAA GGA AAG CCA GCA TCA GA
Klotho	fwd.	CAA CCT CTC GTC TCT TCT GC
	rev.	TGG GAA CTT CAT GTT AGG
Tbp	fwd.	ACC CTT CAC CAA TGA CTC CTA TG
	rev.	TGA CTG CAG CAA ATC GCT TGG
Trpv5	fwd.	CCA CAG TGA TGC TGG AGA GG
	rev.	GGA TTC TGC TCC TGG TGG TG

Table S4. List of primers used for qRT-PCR in the present study

Slc12a3

Gene	rho	p-value
Ppp1r1a	0.5637	2.00E-06
Wnk1	0.5631	2.00E-06
Abca13	0.5306	2.00E-06
Klhl3	0.4893	2.00E-06
Fth1	0.4727	2.00E-06
Uroc1	0.4618	2.00E-06
Sgms2	0.4564	2.00E-06
Fabp3	0.4502	2.00E-06
Lhx1	0.4464	2.00E-06
Egf	0.4408	2.00E-06

Pvalb

Gene	rho	p-value
Ppp1r1a	0.3928	2.00E-06
Fth1	0.3860	2.00E-06
Egf	0.3709	2.00E-06
Wfdc15b	0.3692	2.00E-06
Ckb	0.3447	2.00E-06
Fxyd2	0.3424	2.00E-06
Uqcrq	0.3195	2.00E-06
Gpx6	0.3164	2.00E-06
Uroc1	0.3083	2.00E-06
Cox20	0.3050	2.00E-06

Trpv5

Gene	rho	p-value
S100g	0.3956	2.00E-06
Calb1	0.3818	2.00E-06
Ptges	0.3806	2.00E-06
Clu	0.3642	2.00E-06
Scnn1g	0.3616	2.00E-06
Spp1	0.3390	2.00E-06
Tspan8	0.3235	2.00E-06
Klk1b22	0.3217	2.00E-06
Hsd11b2	0.3189	2.00E-06
Tspan1	0.3185	2.00E-06

Aqp2

Gene	rho	p-value
Hsd11b2	0.5355	2.00E-06
Kcne1	0.4746	2.00E-06
Gm38392	0.4628	2.00E-06
Rhcg	0.4490	2.00E-06
Rhbg	0.4436	2.00E-06
Scnn1g	0.4420	2.00E-06
Aqp3	0.4273	2.00E-06
Cdh16	0.4230	2.00E-06
Tbck	0.4082	2.00E-06
Spink8	0.4067	2.00E-06

Table S5. scRNA-seq data: Gene expression correlation with cell type markers based on correlation analysis with known positive markers. For each positive marker (*Slc12a3*, *Pvalb*, *Trpv5* and *Aqp2*) the 10 most significant correlating genes are listed above.

Correl	Genes	rho	p.value	FDR
Slc12a3	Ppp1r1a	0.5637	2.00E-06	0.002447068
	Wnk1	0.5631	2.00E-06	0.002447068
	Abca13	0.5306	2.00E-06	0.002447068
	Klhl3	0.4893	2.00E-06	0.002447068
	Fth1	0.4727	2.00E-06	0.002447068
	Uroc1	0.4618	2.00E-06	0.002447068
	Sgms2	0.4564	2.00E-06	0.002447068
	Fabp3	0.4502	2.00E-06	0.002447068
	Lhx1	0.4464	2.00E-06	0.002447068
	Egf	0.4408	2.00E-06	0.002447068
	Sfrp1	0.4296	2.00E-06	0.002447068
	Trpm7	0.4254	2.00E-06	0.002447068
	Fxyd2	0.3993	2.00E-06	0.002447068
	Ier3	0.3841	2.00E-06	0.002447068
	Gm37376	0.3514	2.00E-06	0.002447068
	Atp5g1	0.3445	2.00E-06	0.002447068
	Cwh43	0.3431	2.00E-06	0.002447068
	Ptgfr	0.3370	2.00E-06	0.002447068
	Mt1	0.3310	2.00E-06	0.002447068
	Emx1	0.3212	2.00E-06	0.002447068
	Cox6c	0.3075	2.00E-06	0.002447068
	Atp5l	0.2987	2.00E-06	0.002447068
	Gm2065	0.2979	2.00E-06	0.002447068
	Sall3	0.2977	2.00E-06	0.002447068
	Slc5a3	0.2938	2.00E-06	0.002447068
	Cox20	0.2871	2.00E-06	0.002447068
	Larp1b	0.2856	2.00E-06	0.002447068
	Gabarapl1	0.2836	2.00E-06	0.002447068
	Kng2	0.2825	2.00E-06	0.002447068
	Wfdc15b	0.282	2.00E-06	0.002447068

Correl	Genes	rho	p.value	FDR
Pvalb	Ppp1r1a	0.3928	2.00E-06	0.002447068
	Fth1	0.3860	2.00E-06	0.002447068
	Egf	0.3709	2.00E-06	0.002447068
	Wfdc15b	0.3692	2.00E-06	0.002447068
	Ckb	0.3447	2.00E-06	0.002447068
	Fxyd2	0.3424	2.00E-06	0.002447068
	Uqcrq	0.3195	2.00E-06	0.002447068
	Gpx6	0.3164	2.00E-06	0.002447068
	Uroc1	0.3083	2.00E-06	0.002447068
	Cox20	0.3050	2.00E-06	0.002447068
	Umod	0.3010	2.00E-06	0.002447068
	Sdhb	0.2959	2.00E-06	0.002447068
	Ndufa4	0.2912	2.00E-06	0.002447068
	Ndufs8	0.2884	2.00E-06	0.002447068
	Gabarapl1	0.2832	2.00E-06	0.002447068
	Gm10252	0.2830	2.00E-06	0.002447068
	Ftl1	0.2783	2.00E-06	0.002447068
	Lhx1	0.2660	2.00E-06	0.002447068
	Atp5g1	0.2650	2.00E-06	0.002447068
	Ftl1-ps1	0.2585	4.00E-06	0.003472713
	Ndufb8	0.2542	4.00E-06	0.003472713
	Ndufb11	0.2534	4.00E-06	0.003472713
	Fabp3	0.2509	4.00E-06	0.003472713
	Ptgfr	0.2504	4.00E-06	0.003472713
	Gm20538	0.2471	1.20E-05	0.009782147
	Rab27a	0.246	1.40E-05	0.010383125
	Atp5b	0.2452	1.40E-05	0.010383125
	Slc12a3	0.2421	1.80E-05	0.012829726
	Mpc1	0.242	1.80E-05	0.012829726
	Scgb1b18	0.2415	2.00E-05	0.013722369

Correl	Genes	rho	p.value	FDR
Trpv5	S100g	0.3956	2.00E-06	0.002447068
	Calb1	0.3818	2.00E-06	0.002447068
	Ptges	0.3806	2.00E-06	0.002447068
	Clu	0.3642	2.00E-06	0.002447068
	Scnn1g	0.3616	2.00E-06	0.002447068
	Spp1	0.3390	2.00E-06	0.002447068
	Tspan8	0.3235	2.00E-06	0.002447068
	Klk1b22	0.3217	2.00E-06	0.002447068
	Hsd11b2	0.3189	2.00E-06	0.002447068
	Tspan1	0.3185	2.00E-06	0.002447068
	Kl	0.3183	2.00E-06	0.002447068
	Klk1	0.3134	2.00E-06	0.002447068
	Klk1b5	0.3111	2.00E-06	0.002447068
	Gm42793	0.3111	2.00E-06	0.002447068
	Rhcg	0.3103	2.00E-06	0.002447068
	Ltbp3	0.3079	2.00E-06	0.002447068
	Cab39l	0.3077	2.00E-06	0.002447068
	Cav2	0.3032	2.00E-06	0.002447068
	Klk1b4	0.3017	2.00E-06	0.002447068
	Selenof	0.2977	2.00E-06	0.002447068
	Wfdc2	0.2965	2.00E-06	0.002447068
	Atp6v1a	0.2951	2.00E-06	0.002447068
	Cdo1	0.2938	2.00E-06	0.002447068
	Cnih1	0.2925	2.00E-06	0.002447068
	Cdh16	0.2874	2.00E-06	0.002447068
	Rhbg	0.2871	2.00E-06	0.002447068
	Snap47	0.2853	2.00E-06	0.002447068
	Klk1b9	0.2832	2.00E-06	0.002447068
	Rnasek	0.2795	2.00E-06	0.002447068
	Gm38392	0.2791	2.00E-06	0.002447068

Correl	Genes	rho	p.value	FDR
Aqp2	Hsd11b2	0.5355	2.00E-06	0.002447068
	Kcne1	0.4746	2.00E-06	0.002447068
	Gm38392	0.4628	2.00E-06	0.002447068
	Rhcg	0.4490	2.00E-06	0.002447068
	Rhbg	0.4436	2.00E-06	0.002447068
	Scnn1g	0.4420	2.00E-06	0.002447068
	Aqp3	0.4273	2.00E-06	0.002447068
	Cdh16	0.4230	2.00E-06	0.002447068
	Tbck	0.4082	2.00E-06	0.002447068
	Spink8	0.4067	2.00E-06	0.002447068
	Scnn1b	0.4061	2.00E-06	0.002447068
	Tmem45b	0.3919	2.00E-06	0.002447068
	Wfdc2	0.3847	2.00E-06	0.002447068
	Tmsb4x	0.3808	2.00E-06	0.002447068
	Cav2	0.3692	2.00E-06	0.002447068
	Pdzk1ip1	0.3691	2.00E-06	0.002447068
	Arhgap4	0.3674	2.00E-06	0.002447068
	Car2	0.3673	2.00E-06	0.002447068
	Tspan8	0.3636	2.00E-06	0.002447068
	Cdo1	0.3582	2.00E-06	0.002447068
	Krt7	0.3511	2.00E-06	0.002447068
	Fxyd4	0.3491	2.00E-06	0.002447068
	Adgrg1	0.3481	2.00E-06	0.002447068
	Atp6ap2	0.3359	2.00E-06	0.002447068
	Apela	0.3349	2.00E-06	0.002447068
	Tspan1	0.3338	2.00E-06	0.002447068
	Smim5	0.3333	2.00E-06	0.002447068
	Ptges	0.3327	2.00E-06	0.002447068
	Ahnak	0.3303	2.00E-06	0.002447068
	Cav1	0.3237	2.00E-06	0.002447068

Table S6. Top 20 correlating genes with positive markers of DCT and PC-CNT cells. Each list shows genes that significantly correlate with the gene of interest (red). Values provided: rho = correlation value, p-value and FDR (false detection rate).

	Ctrl male		KI-KO^{DC} male			Ctrl female		KI-KO^{DC} female		
Blood parameter	Mean	S.D.	Mean	S.D.	p-value	Mean	S.D.	Mean	S.D.	p-value
pH	7.41	0.01	7.41	0.01	0.66	7.42	0.01	7.41	0.01	0.36
pCO2 [kPa]	3.34	0.15	3.83	0.20	0.10	3.74	0.25	4.00	0.20	0.43
pO2 [kPa]	6.80	0.50	7.10	0.32	0.62	7.08	0.66	7.60	0.37	0.49
HCO3- [mmol/L]	19.28	0.56	20.08	0.42	0.09	20.15	0.76	20.56	0.40	0.63
Na^+ [mmol/L]	147.50	0.05	148.00	0.02	0.29	149.50	0.50	148.40	0.51	0.17
K^+ [mmol/L]	4.48	0.26	4.25	0.09	0.40	4.27	0.12	4.18	0.08	0.53
Ca^{2+} [mmol/L]	1.20	0.02	1.24	0.01	0.15	1.21	0.01	1.23	0.02	0.39
Cl^- [mmol/L]	108.25	0.48	108.60	0.24	0.51	109.25	1.38	107.60	0.51	0.26
Agap [mmol/L]	23.85	0.70	21.86	0.49	0.05	22.28	0.23	22.14	0.57	0.85
Hct [%]	38.25	2.32	38.60	0.81	0.88	39.50	0.71	40.60	0.51	0.06

Table S7. Blood parameters in control and KI-KO^{DC} mice (n=4-5 mice, each group).

	Ctrl		KI-KO^{Kidney}		
Blood parameter	Mean	S.D.	Mean	S.D.	p- value
pH	7.35	0.004	7.30	0.020	0.039
pCO2 [kPa]	5.83	0.26	7.39	0.76	0.293
pO2 [kPa]	7.52	0.46	7.82	0.21	0.674
HCO3- [mmol/L]	23.39	0.81	27.33	1.97	0.006
Na^+ [mmol/L]	145.88	1.59	145.25	0.63	0.114
K^+ [mmol/L]	4.56	0.18	4.90	0.16	0.001
Ca^{2+} [mmol/L]	1.37	0.03	1.53	0.05	0.013
Cl^- [mmol/L]	109.14	1.13	106.25	1.18	0.002
Agap [mmol/L]	14.00	0.95	12.75	1.46	0.260
Hct [%]	39.86	0.44	42.50	1.27	0.004

Table S8. Blood parameters in control and KI-KO^{Kidney} mice (n=4 male mice, each group).
